# Real-time evaluation of signal accuracy in wastewater surveillance of pathogens with high rates of mutation

**DOI:** 10.1038/s41598-024-54319-y

**Published:** 2024-02-14

**Authors:** Ocean Thakali, Élisabeth Mercier, Walaa Eid, Martin Wellman, Julia Brasset-Gorny, Alyssa K. Overton, Jennifer J. Knapp, Douglas Manuel, Trevor C. Charles, Lawrence Goodridge, Eric J. Arts, Art F. Y. Poon, R. Stephen Brown, Tyson E. Graber, Robert Delatolla, Christopher T. DeGroot, Ontario Wastewater Surveillance Consortium, Adebowale Adebiyi, Matthew Advani, Simininuoluwa Agboola, Dania Andino, Hussain Aqeel, Yash Badlani, Lena Carolin Bitter, Leslie Bragg, Patrick Breadner, David Bulir, Ronny Chan, Babneet Channa, Trevor Charles, JinJin Chen, Ryland Corchis-Scott, Matthew Cranney, Patrick M. D’Aoust, Hoang Dang, Nora Danna, Rachel Dawe, Tomas de Melo, Jean-Paul Desaulniers, Hadi Dhiyebi, Justin Donovan, Elizabeth Edwards, Isaac Ellmen, Joud Abu Farah, Farnaz Farahbakhsh, Meghan Fuzzen, Tim Garant, Qiudi Geng, Ashley Gedge, Alice Gere, Richard Gibson, Kimberly Gilbride, Eyerusalem Goitom, Qinyuan Gong, Marc Habash, Amanda Hamilton, Blake Haskell, Samina Hayat, Nada Hegazy, Hannifer Ho, Yemurayi Hungwe, Heather Ikert, Golam Islam, Dilan Joseph, Ismail Khan, Richard Kibbee, Andrea Kirkwood, Jennifer Knapp, James Knockleby, Su-Hyun Kwon, Christopher Kyle, Opeyemi U. Lawal, Line Lomheim, Robert Michael McKay, Ria Menon, Zach Miller, Aleksandra M. Mloszewska, Ataollah Mohammadiankia, Shiv Naik, Delaney Nash, Anthony Ng, Abayomi Olabode, Banu Örmeci, Claire Oswald, Alyssa Overton, Gabriela Jimenez Pabon, Vinthiya Paramananthasivam, Jessica Pardy, Valeria R. Parreira, Sarah Jane Payne, Hui Peng, Lakshmi Pisharody, Samran Prasla, Melinda Precious, Fozia Rizvi, Matthew Santilli, Hooman Sarvi, Mark Servos, Dan Siemon, Denina Simmons, Carly Sing-Judge, Nivetha Srikanthan, Sean Stephenson, Jianxian Sun, Endang Susilawati, Amir Tehrani, Shen Wan, Martin Wellman, Katie Williams, Ivy Yang, Gustavo Ybazeta, Eli Zeeb

**Affiliations:** 1 Department of Civil Engineering, University of Ottawa, Ottawa, ON K1N 6N5 Canada; 2 Children’s Hospital of Eastern Ontario Research Institute, Ottawa, ON K1H 8L1 Canada; 3 The Ottawa Hospital Research Institute, 1053 Carling Ave, Ottawa, ON K1Y 4E9 Canada; 4 Department of Biology, University of Waterloo, 200 University Avenue West, Waterloo, ON N2L 3G1 Canada; 5 Department of Family Medicine, University of Ottawa, 75 Laurier Ave. E, Ottawa, ON K1N 6N5 Canada; 6 School of Epidemiology and Public Health, University of Ottawa, 75 Laurier Ave. E, Ottawa, ON K1N 6N5 Canada; 7 Department of Food Science, Canadian Research Institute for Food Safety, University of Guelph, Guelph, ON N1G 2W1 Canada; 8 Department of Microbiology and Immunology, Western University, London, ON N6A 3K7 Canada; 9 School of Environmental Studies and Department of Chemistry, Queen’s University, Kingston, ON Canada; 10 Department of Mechanical and Materials Engineering, Western University, London, ON N6A 5B9 Canada; 11 Department of Chemistry, University of Toronto, 80 St. George St., Toronto, ON M5S 3H6 Canada; 12 Department of Chemical Engineering and Applied Chemistry, University of Toronto, 200 College St., Toronto, ON M5S 3E5 Canada; 13 Health Sciences North Research Institute, 56 Walford Rd., Sudbury, ON P3E 2H2 Canada; 14 Department of Chemistry and Biology, Toronto Metropolitan University, 450 Victoria St., Toronto, ON M5B 2K3 Canada; 15 Department of Civil and Environmental Engineering, Carleton University, 1125 Colonel By Dr., Ottawa, ON K1S 5B6 Canada; 16 Department of Pathology and Molecular Medicine, McMaster University, 1200 Main St. W., Hamilton, ON L8S 4K1 Canada; 17 Great Lakes Institute for Environmental Research, University of Windsor, 401 Sunset Ave., Windsor, ON N9B 3P4 Canada; 18 Faculty of Sciences, Ontario Tech University, 2000 Simcoe St. N., Oshawa, ON L1G 0C5 Canada; 19 Department of Geography and Environmental Studies, Toronto Metropolitan University, 350 Victoria St., Toronto, ON M5B 2K3 Canada; 20 School of Environmental Sciences, University of Guelph, 50 Stone Rd. E., Guelph, ON N1G 2W1 Canada; 21 Forensic Science Department, Trent University, 1600 West Bank Dr., Peterborough, ON K9L 0G2 Canada; 22 Department of Civil Engineering, Queen’s University, Kingston, ON K7L 3N6 Canada

**Keywords:** Viral infection, RNA sequencing, Microbiology techniques

## Abstract

Wastewater surveillance of coronavirus disease 2019 (COVID-19) commonly applies reverse transcription-quantitative polymerase chain reaction (RT-qPCR) to quantify severe acute respiratory syndrome coronavirus 2 (SARS-CoV-2) RNA concentrations in wastewater over time. In most applications worldwide, maximal sensitivity and specificity of RT-qPCR has been achieved, in part, by monitoring two or more genomic loci of SARS-CoV-2. In Ontario, Canada, the provincial Wastewater Surveillance Initiative reports the average copies of the CDC N1 and N2 loci normalized to the fecal biomarker pepper mild mottle virus. In November 2021, the emergence of the Omicron variant of concern, harboring a C28311T mutation within the CDC N1 probe region, challenged the accuracy of the consensus between the RT-qPCR measurements of the N1 and N2 loci of SARS-CoV-2. In this study, we developed and applied a novel real-time dual loci quality assurance and control framework based on the relative difference between the loci measurements to the City of Ottawa dataset to identify a loss of sensitivity of the N1 assay in the period from July 10, 2022 to January 31, 2023. Further analysis via sequencing and allele-specific RT-qPCR revealed a high proportion of mutations C28312T and A28330G during the study period, both in the City of Ottawa and across the province. It is hypothesized that nucleotide mutations in the probe region, especially A28330G, led to inefficient annealing, resulting in reduction in sensitivity and accuracy of the N1 assay. This study highlights the importance of implementing quality assurance and control criteria to continually evaluate, in near real-time, the accuracy of the signal produced in wastewater surveillance applications that rely on detection of pathogens whose genomes undergo high rates of mutation.

## Introduction

Reverse transcription quantitative polymerase chain reaction (RT-qPCR)-based detection of severe acute respiratory syndrome coronavirus 2 (SARS-CoV-2) RNA is the commonly applied diagnostic test for coronavirus disease 2019 (COVID-19) in humans. As of July 2023, the same nucleic acid amplification-based approach has been applied to nearly 4500 locations across the world for wastewater surveillance (WWS) of COVID-19, according to the COVIDPoops19 dashboard^1^. RT-qPCR commonly uses primers and a TaqMan hydrolysis probe that are designed to bind to specific regions of the viral cDNA (reverse-transcribed viral RNA), enabling the detection of RNA fragments of the target organism with high specificity in both human and wastewater samples. Due to the complex nature of wastewater, which promotes degradation of SARS-CoV-2 RNA, use of assays that target multiple genetic loci has been recommended for WWS of SARS-CoV-2^2^. In this regard, the United States Centers for Disease Control and Prevention (CDC) N1 and N2 RT-qPCR assays that amplify regions of the SARS-CoV-2 nucleocapsid (N) gene are widely used for WWS due to their high sensitivity and specificity observed early in the pandemic^3^.

SARS-CoV-2, like all coronaviruses, accumulates mutations quickly due to virus-intrinsic as well as epidemiological factors. In particular, this virus encodes a (600–700 nts/s) viral RNA-dependent RNA polymerase (RdRp) that lacks proofreading activity, further enhancing mutations. Although the exonuclease activity of the nsp14-nsp10 proteins maintain some level of transcriptional fidelity, it is inherently low^4^. Intrinsically high mutation rates are compounded by recombination events, ostensibly arising from co-infection of the same host cell, and the high transmission and global prevalence of infections only exacerbates viral genetic diversity. In view of possible mutations that can result in increased virulence, transmissibility, and immune escape from antibodies, efforts have been made globally to understand the evolutionary dynamics of SARS-CoV-2^5^. As of April 24, 2021, 115 mutations with a prevalence rate of more than 3% had been identified by analyzing over one million SARS-CoV-2 sequences from 98 countries^6^. With the emergence of SARS-CoV-2 variants, the utmost concern for WWS is mutations in regions that produce primer/probe sequence mismatches; thus, having the potential to decrease the sensitivity and/or specificity of the assay and, in particular, the commonly applied CDC N1 and N2 RT-qPCR assays^7^. Nucleotide mismatches in primers can affect annealing to their complementary target sequence, reducing amplification^8^. In addition, mismatches in the probe can affect exonuclease activity of the Taq polymerase, causing reduction in fluorescence intensity^9^. These effects of nucleotide mismatches can result in false negative and/or underestimation of targets when using RT-qPCR.

Considering the emergence of different SARS-CoV-2 variants, WWS has been utilized in tracking variants using allele-specific RT-qPCR (AS-RT-qPCR) in addition to high throughput sequencing^2,10–16^. Both methods have their own sets of advantages and challenges when applied to wastewater samples. Sequencing provides a comprehensive understanding of the genome but is time-consuming, resource-intensive, and can be affected by low coverage when dealing with environmental samples, thereby requiring considerable optimization. Meanwhile, AS-RT-qPCR has a quick turnaround time but cannot detect novel variants, requires validation of sensitivity and specificity of each assay for each mutation, and can require multiple PCR runs to test for the constellation of mutations to diagnose variants of concern. Due to the various advantages and challenges of the above-mentioned methods, the Wastewater Surveillance Initiative (WSI) in the Canadian province of Ontario has applied both AS-RT-qPCR and genomic sequencing to track variants in the wastewater in addition to almost all sites applying both N1 and N2 gene region assays for surveillance of SARS-CoV-2 levels in wastewater. During the period of study, the WSI program included 175 sampling locations across the province, including both wastewater treatment facilities and sites within the sewer network. In sum, approximately 75% of the ~ 15 M population of Ontario was represented by the sampling network^17^. The analyses were performed by 13 different universities/institutions, namely: Carleton University, University of Guelph, Health Science North Research Institute, McMaster University, University of Ontario Institute of Technology, University of Ottawa, Queens University at Kingston, University of Toronto, Toronto Metropolitan University, Trent University, University of Waterloo, University of Windsor, and Western University.

Despite the implementation of WWS across Ontario and numerous countries worldwide, there still exists a lack of guidelines on standards of practice for SARS-CoV-2 WWS by the scientific community, as this is still an emerging field. This has resulted in a critical need for operating laboratories to implement in-house, robust quality assurance/quality control (QA/QC) of their data to identify erroneous results that can mislead public health responses. The quantitative dual loci QA/QC criteria developed and implemented by the University of Ottawa in partnership with the Children’s Hospital of Eastern Ontario Research Institute includes a comparison of the N1 and N2 gene targets for SARS-CoV-2 WWS to identify assay deficiencies such as reagent quality and laboratory errors. The QA/QC criterion is also able to identify the mechanistic loss of sensitivity of assays due to the onset of new mutations in the assay primer/probe gene regions in one of the two assays implemented. If the loss of sensitivity is found to be significant, this can lead to a timely assay modification to maintain accuracy of the surveillance protocol which will ensure that correlation with public health metrics (e.g. clinical cases or hospitalizations) is maintained.

Herein we report the underestimation of SARS-CoV-2 by CDC N1 when compared with N2 assays between July 9, 2022, and January 31, 2023, in the Ontario WSI program, identified through the implementation of a novel, quantitative dual loci QA/QC criterion. The main objective of this study is to demonstrate how a combination of robust QA/QC criteria accompanying near real-time RT-qPCR for SARS-CoV-2 identified a loss of sensitivity in implemented methods and the use of allele-specific RT-qPCR (AS-RT-qPCR) and sequencing methods identified the variant driving the loss of sensitivity during the onset of a new COVID-19 wave. This study highlights the need for rigorous QA/QC criteria and the use of multiple tools for WWS of SARS-CoV-2. Lessons learned from this study will guide future studies in disease surveillance involving pathogens with high mutation rates.

## Materials and methods

### QA/QC criteria

The QA/QC criteria implemented at the University of Ottawa includes two dual loci criteria that were responsible for early identification of the loss of sensitivity in the CDC N1 assay based on samples from the City of Ottawa. The first criterion requires that the magnitude of the difference between N1 and N2 gene copies, normalized by the average of the N1 and N2 gene copies, must be less than or equal 0.5:

1 $$\frac{|N1-N2|}{\frac{1}{2}\left(N1+N2\right)}\le 0.5$$

The criterion, based on the relative difference, was established from the analysis of 156 paired biological replicates collected from seven unique sampling locations. This criterion was implemented on October 29, 2021, and its validity was confirmed with over 630 samples from the same seven locations, as well as two additional sites added later, before the commencement of this study. The following additional QA/QC criteria, based on the MIQE guidelines for RT-qPCR^18,19^ were implemented in addition to the above criterion for all samples analyzed: (i) no amplification in negative template control (NTC) or extraction blank; (ii) the standard deviation of technical triplicate *C*_t_ values is < 0.5 (iii) the cycle delay between the dilution points must be within one *C*_t_ of the expected value, indicating absence of PCR inhibition, and (iv) the standard curve has a coefficient of determination, *r*^2^, > 0.98, a y-intercept between 37.0 and 39.5, and a slope between, − 3.1 and − 3.6 (i.e. PCR efficiency from 90 to 110%).

A Z-score metric was also implemented to quantify the deviation between the N1 and N2 assays. This metric was monitored in real-time for the City of Ottawa and retrospectively for all other sites in the provincial program measuring both N1 and N2. The Z-score represents how many standard deviations from the mean a certain observation lies, and is defined as:

2 $$\text{Z-score} =\frac{x-\mu }{\sigma }$$

where:

3 $$x=\frac{N1-N2}{N1+N2}$$

and $\mu$ and $\sigma$ are the mean and standard deviation, respectively, of the quantity $x$ over the time period of interest. For N1 and N2 assays with equal accuracy, the mean value of $x$ is expected to be close to zero. As $x$ represents the error between the two signals, it is assumed that it is well approximated as a normal distribution. Also note that $x$ resembles the left side of the relative difference criterion (Eq. 1), except for the factor ½. Since this factor would appear in both the numerator and denominator of Eq. (2), it can be omitted in the calculation of the Z-score.

### Wastewater surveillance of SARS-CoV-2 by RT-qPCR

Daily 24-h composite primary clarified sludge samples were collected from the City of Ottawa water resource recovery facility and analyzed for SARS-CoV-2 genetic material with a processing time of 8 h and turnaround reporting time of 24 h. The samples were processed as previously described by D’Aoust et al.^20,21^ and were reported to the WSI program and Ottawa Public Health (https://613covid.ca/wastewater/). The CDC N1 and N2 SARS-CoV-2 genomic regions^22^ and the replication-associated protein encoding region of the pepper mild mottle virus (PMMoV)^23,24^ were quantified on a Bio-Rad CFX96 thermal cycler (Bio-Rad Laboratories, Hercules, CA, USA). Both the CDC N1 and N2 RT-qPCR mixtures contained 2.5 µL of TaqMan™ Fast Virus 1-Step RT-PCR Master Mix (Thermo Fisher Scientific, Ottawa, Canada), forward and reverse primers with a concentration of 500 nM, probe concentration of 125 nM (2019-nCoV RUO Kit, Integrated DNA Technologies, Kanata, Canada), 3 µL of template RNA, and 3.75 µL of Rnase-free water. Thermal conditions included reverse transcription at 50 °C for 5 min, initial denaturation at 95 °C for 20 s, followed by 45 cycles of 95 °C for 3 s and 60 °C for 30 s. Five-point standard curves were generated using an RNA standard procured from Exact Diagnostics (Bio-Rad Laboratories, Fort Worth, USA). The same standard was always used simultaneously for both the N1 and N2 assay and the no-template controls were comprised of Rnase-free water.

### Quantifying variants of concern by AS-RT-qPCR

AS-RT-qPCR assays targeting general Omicron viral fragments and distinct mutations to differentiate Omicron sublineages were developed in this study and summarized in Table S1. Optimized AS-RT-qPCR mixture included TaqMan™ Fast Virus 1-Step RT-PCR Master Mix (Thermo Fisher Scientific, Ottawa, Canada), forward and reverse primers (final concentration of 800 nM), probe (final concentration of 250 nM), and Rnase-free water. The temperature profile used for AS-RT-qPCR was as follows: 50 °C for 10 min, 95 °C for 2 min, followed by 45 cycles of 95 °C for 5 s and 58 °C for 40 s. All RT-qPCR sample plates were run in triplicate with no-template controls and gBlock dsDNA fragments (Table S2) were used to generate standard curves as previously reported^11,25^. Samples were considered positive if at least 2 of 3 PCR replicates reported with a C_t_ < 40. Assay performance parameters including slope, y-intercept values, and *R*^2^ values were derived from the mean of 5-point standard curves run on each sample plate and are summarized in Table S3. The proportion of wastewater signal attributable to each allele was calculated as a fraction of the sum of allelic copies determined from the sample plate’s standard curve^11^. For example, the frequency of S:K444T allele was determined with the following equation:

4 $$\% S:K444T=\frac{copies (S:K444T)}{copies (S:K444+S:K444T)}$$

In the case of the S:L452 locus, where three alleles were present in Spring 2022, all three alleles were measured (S:L452, S:L452Q, S:L452R) and their sum represented in the denominator. During the study period, additional mutations arose in the primer/probe loci that required new assays to be created to monitor the S:L452 and S:K444 loci. These new assays are denoted by “0.2” (e.g., S:L452R.2).

### Quantifying variants of concern by genomic sequencing

One raw influent wastewater sample collected weekly from the City of Ottawa was sent for tiled amplicon sequencing at the University of Waterloo. In preparation for sequencing, the viral content of the sample was concentrated using Nanotrap Microbiome A Particles (Ceres Nano, Manassas, USA) and the RNA was extracted using an automated Rneasy Mini Kit (Qiagen, Germantown USA). RNA was then reverse transcribed using LunaScript RT SuperMix Kit (New England Biolabs, Ipswitch, USA) and amplified using Q5^®^ High-Fidelity 2X Master Mix (New England Biolabs, Ipswitch, USA) and the Artic V4.1 NCOV-2019 panel of primers (Integrated DNA Technologies, Kanata, Canada) resulting in ~ 400 bp amplicons. Amplicon libraries were prepared using the Illumina DNA Library Prep kit (20060059) (Illumina, San Diego, USA) with an additional 0.8X AMPure XP (Beckman Coulter, Brea, USA) single sided bead cleanup to remove primer dimers and purify amplicons prior to tagmentation. Finished libraries were run on a MiSeq using V2 500 cycle kits (Illumina, San Diego, USA). Similar methods were used for selected samples at other sites within the provincial WSI network and were sequenced at one of the three sequencing labs, which included the University of Guelph, the University of Waterloo, and Western University.

Data output in the form of FASTQ files were analyzed using a custom pipeline (https://github.com/PoonLab/gromstole) which employs minimap2^26^ to map reads to the SARS-CoV-2 reference genome (Genbank accession NC_045512.2) and generates coverage and mutation frequency data at all sites along the genome. Lineage frequencies for the City of Ottawa were generated and visualized using ALCOV^27^.

### Statistical analysis

The Shapiro–Wilk test, Mann–Whitney test, Loess and Spline regression were carried out with GraphPad Prism version 9.5.0 (GraphPad Software, California, USA). The Z-score for the City of Ottawa data was calculated using Microsoft Office Excel 2019 (Microsoft Corporation, WA, USA). The Z-score and analysis for the provincial data was conducted using Python scripts utilizing the numpy, pandas, and matplotlib libraries.

## Results

### Robust CDC N1 and N2 signal accuracy in Ottawa WWS prior to and following the emergence of Omicron [prior to Omicron: April 8, 2020, to November 28, 2021; early Omicron: November 29, 2021, to July 9, 2022]

**Figure 1:**
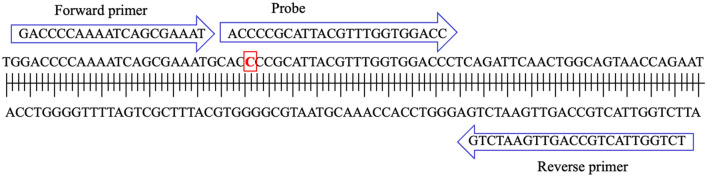
Schematic layout of the CDC N1 primers and probe sequence. The red rectangle indicates the position of C28311T mutation that also corresponds to the third nucleotide position from the 5’ end of the probe region of N1 assay. Nucleotide coordinates are derived from Genbank SARS-CoV-2 reference sequence NC_045512.

SARS-CoV-2 RNA was quantified by monitoring two genomic loci located in the N open reading frame by RT-qPCR (using the CDC N1 and N2 assays) and their average signal was reported as a part of the Ontario WSI. The two-locus approach increased accuracy by decreasing the chance of false positive detection (i.e., by increasing specificity). It also provided a mechanism by which to monitor signal accuracy, should mutation or fragmentation occur at one locus; this would become apparent as a change of signal relative to the unaffected locus. While both the N1 and N2 assay loci were largely unaffected by mutation in the first year of the pandemic, the advent of Omicron in November 2021, raised concerns of loss of sensitivity of the CDC N1 assay as B.1.1.529 genomes contained a C28311T mutation that corresponded to the third nucleotide position from the 5′ end of the DNA probe of the CDC N1 assay (Fig. 1).

The deviation between the N1 and N2 signals in the daily Ottawa WWS samples was assessed using the two methods described in the “QA/QC criteria” section, namely the relative difference criterion and the Z-score during a monitoring period starting from April 8, 2020. It was observed that the relative difference criterion was exceeded on 36 occasions, never more than two days in a row, across a period of 479 consecutive days starting April 8, 2020, until the first reported Omicron case in Ontario, Canada on November 28, 2021. During the period of early period Omicron in Ontario, between November 28, 2021, and July 9, 2022 (where BA.1*, BA.2*, BA.4*, BA.5*, all harbouring the C28311T mutation, were the dominant lineages), the relative difference criterion was exceeded on 14 occasions over a period of 221 days. During this time Z-scores were used to retrospectively assess the directionality and trend in the relative abundance of N1 and N2 and was then implemented as a new ongoing QA/QC criterion by the University of Ottawa. Negative Z-scores indicate a degradation of N1 signal, or alternatively, enhanced N2 signal. The mean and standard deviation used in the definition of the Z-score were based on the period from April 8, 2020, up until the present time such that the real-time trend could be assessed. During this period, the deviation of the Z-score did not indicate a concerning change in the N1 signal accuracy, as will be shown later. Therefore, during the early emergence of Omicron, the infrequent failure of the relative difference criterion and the real-time trend in the Z-score were considered indicative of robust signal accuracy during this period, despite the presence of the C28311T mutation in the CDC N1 probe region.

### Loss of signal accuracy in Ottawa WWS concomitant with the emergence of Omicron sub-lineages with an additional mutation in the N1 assay probe region [late Omicron: July 10, 2022, to January 31, 2023]

**Figure 2:**
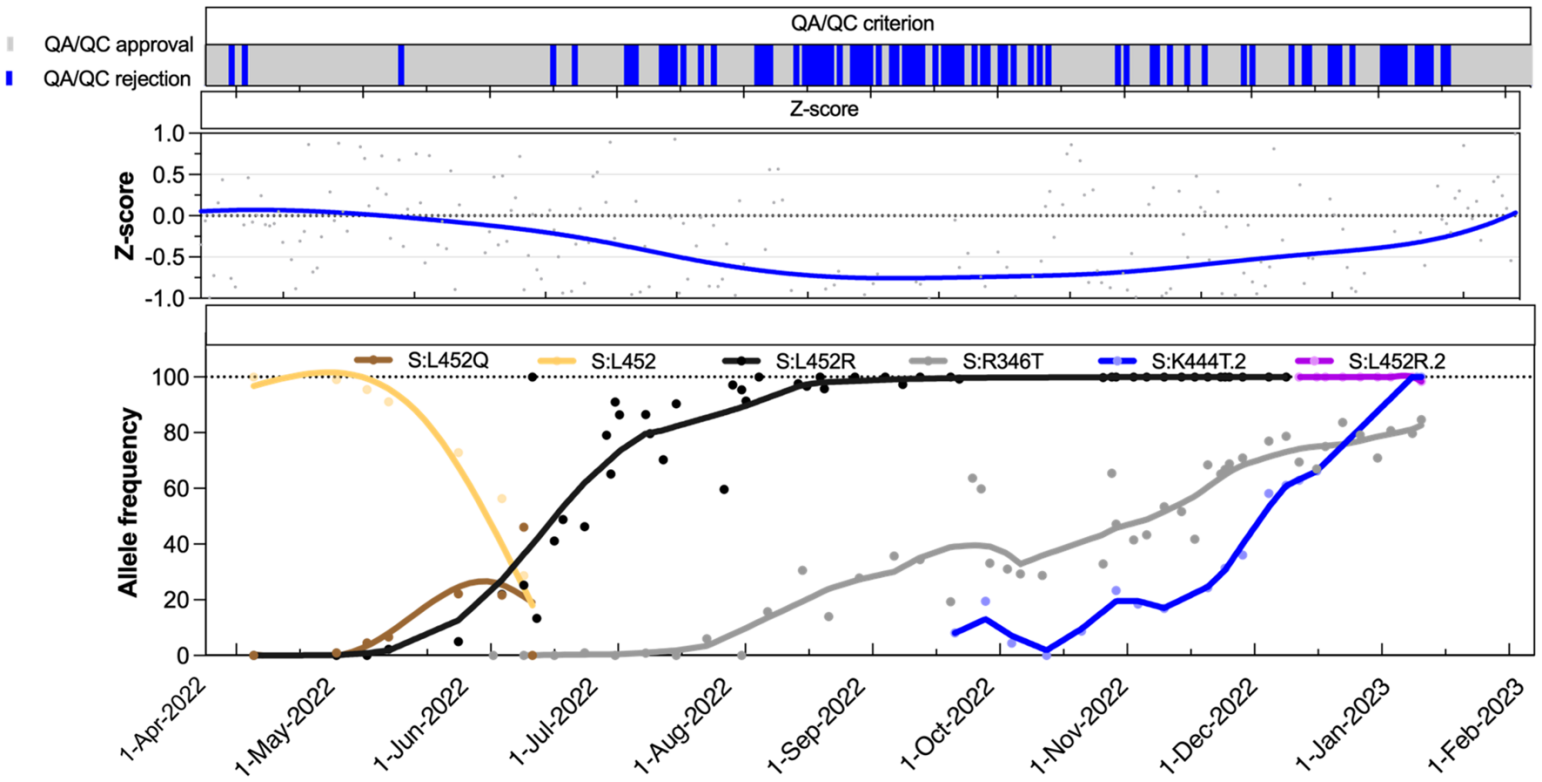
Plot displaying the QA/QC criterion fulfillment, the Z-score, and the proportion of mutations in wastewater across a period of April 1, 2022, to January 31, 2023, in wastewater of Ottawa. The regression curves are Loess regression curve except for S:L452 and S:L452Q for which Spline regression curves are drawn. The frequency estimates are based on AS-RT-qPCR assays.

For the first time, the relative difference criterion between the N1 and N2 signals was exceeded on three consecutive days on July 7, 8, and 9, 2022. Then, the criterion was exceeded 71 times across a period of 205 consecutive days during the daily, real-time monitoring of the wastewater for SARS-CoV-2 signal in Ottawa during the late Omicron period from July 10, 2022, to January 31, 2023. This high frequency of failure, putatively caused by an underestimation of N1 copies, was identified in real-time based on the outlined relative difference and Z-score QA/QC criteria, shown in Fig. 2. The Z-score in Fig. 2 (see Table S4 for Z-score values) was calculated retrospectively based on the period from April 8, 2020, to January 31, 2023, and would therefore differ slightly from the real-time values that were used in the daily QA/QC process, which were calculated up to the most recent data at that time. The Z-score reached its minimum value on October 1, 2022, based on a LOESS regression curve (Fig. 2), which coincided with the high frequency failure of the relative difference criterion. These changes in the Z-score and the increased frequency of failure of the relative difference criterion were considered to likely be indicative of the emergence of new sub-lineages with mutation(s) that significantly compromised the N1 signal accuracy and sensitivity. This enabled rapid communication of this loss of assay sensitivity to relevant stakeholders including processing laboratories and their public health partners.

**Figure 3:**
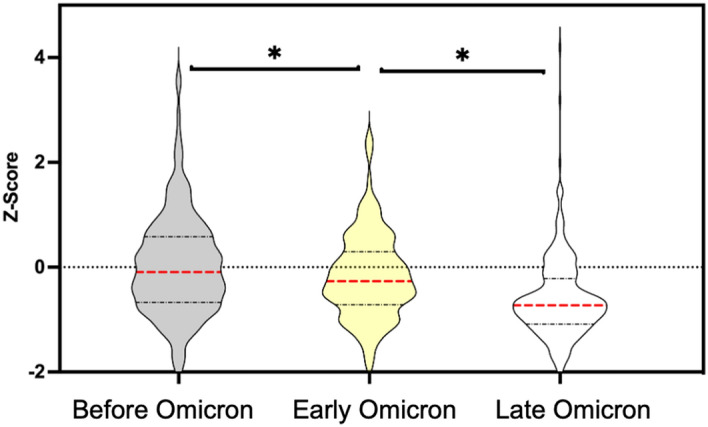
Violin plot showing Z-scores from the Before Omicron (April 8, 2020 to November 28, 2021), Early Omicron (November 29, 2021 to July 9, 2022), and Late Omicron (July 10, 2022 to January 31, 2023) periods. The asterisk (*) indicates significant difference in Z-score values between two groups (Mann–Whitney test; p < 0.05). Red dotted lines indicate median Z-score values, and the black dashed lines indicate the interquartile range.

The retrospectively calculated Z-scores between the April 8, 2020 and January 31, 2023 were binned based on 3 periods: “Before Omicron”, “Early Omicron”, and “Late Omicron” (Fig. 3). Before Omicron (prior to November 28, 2021) represents a period where there were no significant mutations in the N1 probe region. Early Omicron (November 28, 2021 to July 9, 2022) represents a period where all dominant lineages harboured the C28311T mutation. Late Omicron (July 10, 2021 to January 31, 2023) represents a period where the dual loci QA/QC criteria indicated reduced sensitivity of the N1 assay, putatively caused by additional mutations in the CDC N1 probe region. It was found that there is a significant difference in Z-score values (*p* < 0.05, Mann Whitney test) when comparing the Early Omicron period to the Before Omicron period, indicating that the C28311T mutation did significantly affect the accuracy of the N1 assay. However, the interquartile range of the Z-score encompasses zero and the magnitude of the mean Z-score is only around 0.3 (i.e., the mean observation was 0.3 standard deviations away from the mean). Therefore, it is concluded that while the performance of the N1 assay was affected by the presence of the C28311T mutation in the probe region, the signal degradation was not sufficient to consider any changes to data reporting. The Z-score was shown to be statistically different (Mann–Whitney test; *p* < 0.05) in the Late Omicron period compared to the Early Omicron period (Fig. 3), indicative of consistently lower N1 assay signal compared to the N2 assay signal. In this case, the median Z-score is about 0.7 and the interquartile range does not include zero; this raises significant concerns about the accuracy of the N1 assay.

**Figure 4:**
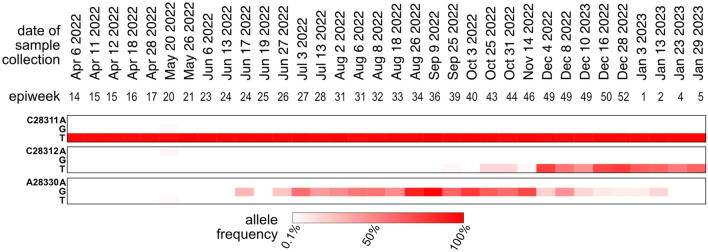
Heatmap of mutation frequency within the CDC N1 probe sequence. The frequency estimates are based on tiled amplicon sequencing of 24-h composited samples of wastewater treatment plant influent in the City of Ottawa.

To test the hypothesis that the change in sensitivity of the N1 assay was due to additional mutations in the N1 probe, mutation frequency estimates from tiled amplicon sequencing were curated, focusing on those loci mapping to the CDC N1 RT-qPCR assay primer and probe regions. These were compiled across multiple sequencing runs per month starting just prior to the introduction of Omicron (Delta waning in November 2021) and ending the first week of February 2023 as BQ-like variants waned and XBB-like variants were emerging in Ottawa wastewater. It is noted that the vast majority of the locus across the entire period was conserved, however, there was a clear and rapid transition to C28311T with the equally rapid emergence of Omicron in Ottawa in early December 2021, as shown in Fig. 4. As expected, this mutation has remained at 100% frequency in wastewater samples until the end of the period. Moreover, increased frequency of A28330G was noted as early as July 3, 2022, consistent with the observation of the negative Z-score trend at that time. The presence of the C28311T/A28330G mutations at > 50% persisted until November 14, 2022, after which the emergence of C28311T/C28312T/A28330G at > 50% was observed in the next sequenced sample of December 4, 2022. These nucleotide-level data match the timing of changes in N1 signal accuracy described and thus confirm that the Z-score faithfully reflects changes in COVID-19 WWS accuracy caused by mismatches between the N1 probe sequence and RNA fragments present in the sample. It is noted that during the time period under consideration there were no significant mutations in the N2 primer/probe regions.

The dominant predicted lineage derived from two samples collected from the Ottawa treatment plant in the first and second week of July 2022 were assigned Pango lineage BA.5.2.1 (Fig. S1), which does not include either the S:R346T or A28330G mutation; however, poor coverage at S:R346 was reported (73 and 13, respectively, for the samples collected July 3 and July 13). During the same period, convergent mutations across an increasing number of GISAID Omicron genomes were being compiled and reported by CoV-Spectrum contributors^5^ including S:R346T. Therefore, AS-RT-qPCR for S:R346T was carried out and was first detected on July 25, 2022. As shown in Fig. 2, subsequently collected samples showed increasing frequency of the S:R346T mutation, along with a further decrease in the Z-score. Taken together, these data support the idea that BF.7 (BA.5.2.1 + S:R346T), or other BF.* sub-lineages that include both the C28311T and A28330G mutations, were the variant(s) mainly responsible for the reduced N1 signal accuracy observed in Ottawa during the period of emergence of Omicron sub-lineages.

### Recovery of sensitivity and accuracy of the N1 assay during the end of the Late Omicron period [July 10, 2022 to January 31, 2023] with the transition between BQ-like and XBB sub-lineages

In October 2022, reports of emergence and rapid spread of BQ-like variants were reported globally^28^. Therefore, BQ-like Omicron sub-lineage-diagnostic S:K444T AS-RT-qPCR assay was designed and deployed to track the spread of BQ-like sub-lineage in wastewater of Ottawa. The S:K444T mutation was first detected on September 20, 2022. A continuous increase in the proportion of S:K444T mutation and positive deflection of Z-score was observed as S:K444T frequency increased past 50% on December 1, 2022 reaching totality on January 3, 2023 (Fig. 2). During the same period, frequency of A28330G was also shown to be less than 10%, whereas C28311T/C28312T persisted at high rates (Fig. 4). This is expected since the A28330G mutation is absent in BQ-like sub-lineages (cov-spectrum.org). Therefore, our findings likely suggest that A28330G was the most significant mutation affecting the accuracy and sensitivity of N1 assay, since the absence of this mutation improved the N1 signal relative to N2.

### Confirmation of mutation effects across the provincial surveillance program

**Figure 5:**
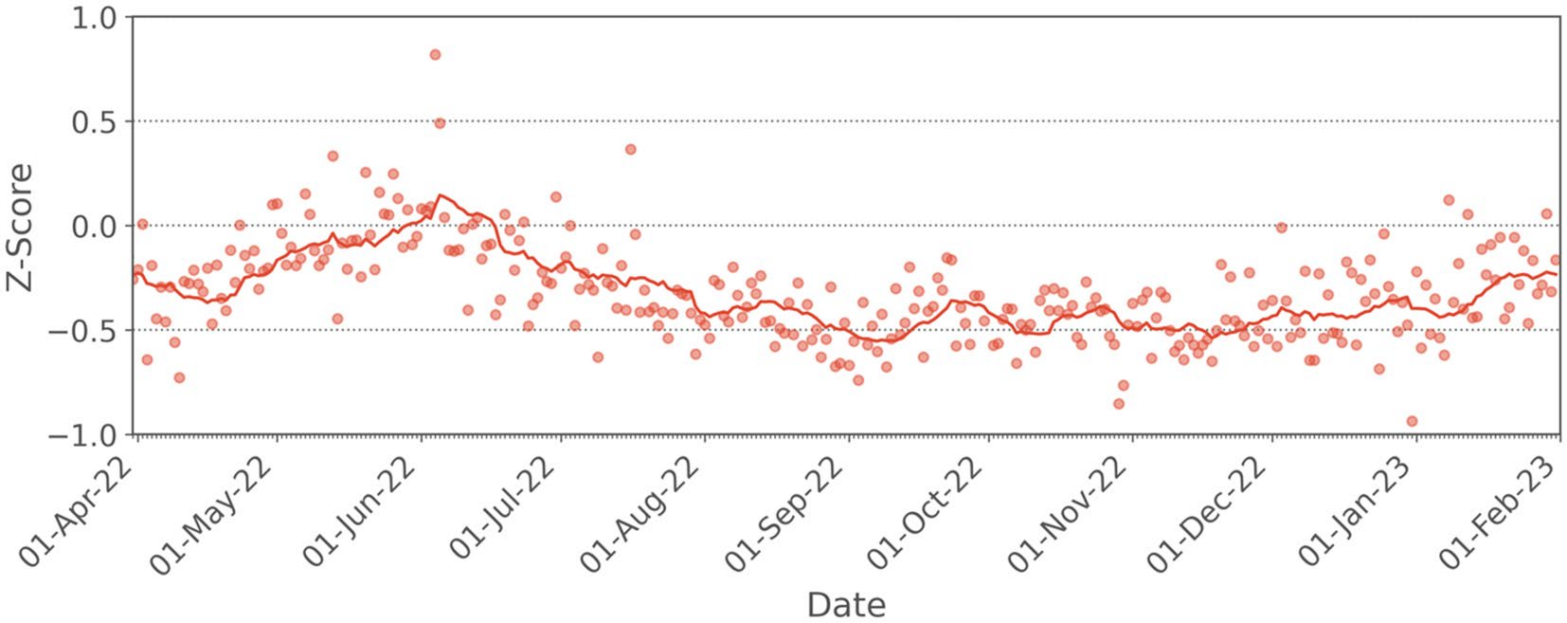
Plot of the Z-score, averaged for each day over all sampling sites in the Ontario WSI. The solid line represents the 14-day moving average.

Of the 13 labs participating in the Ontario WSI program, 11 measured both N1 and N2 gene targets for a minumum of 50% of the time period between April 1, 2022 and January 31, 2023. For these sites, the Z-score was calculated using lab-specific means and standard deviations using all data collected up to and including January 31, 2023. In total 24,196 samples are included in the provincial analysis. Figure 5 shows these data averaged across all samples on a daily basis. Within the period considered, the 14-day moving average of the Z-score remained within an approximate envelope between ± 0.3 until mid-July, 2022; this is consistent with the Ottawa date of July 10 where the emergence of Omicron sub-lineages was assigned due to repeated failure of the dual loci QA/QC criterion. The 14-day moving average remains outside of the ± 0.3 envelope until mid-January, 2023. This is also consistent with the Ottawa data where the Z-score increases toward zero as BQ-like sub-lineages emerged in late 2022 and into 2023.

**Figure 6:**
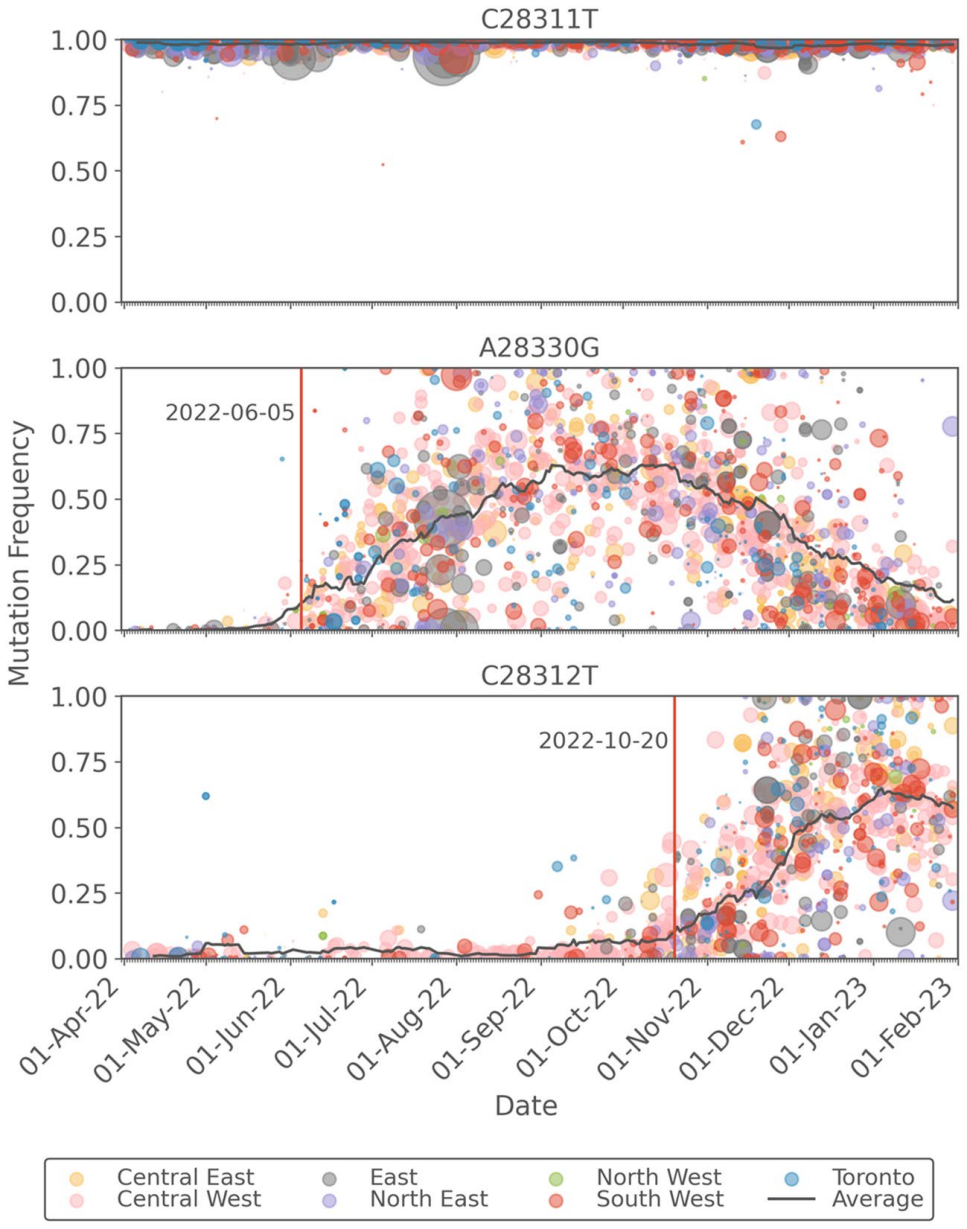
Plot of the frequencies of the C28311T, A28330G, and C28312T mutations which are present in the N1 region of the N gene. Data points are coloured by the Ontario sub-region from which the sample was taken and are sized in proportion to the sequence coverage. The solid black line represents the 14-day moving average of the daily-averaged frequencies across all sampling sites. The vertical red lines indicate the first date at which the moving average exeeds a frequency of 0.1.

A subset of 2147 of the provincial samples analyzed for viral load were also sequenced. These data are separated into seven sub-regions according regional groupings of public health units (Table S5). The frequencies of the key N gene mutations are plotted in Fig. 6, where the colours indicate the sub-region from which the sample originated and the size of each point is proportional to the sequencing coverage (i.e. the average number of reads that align to the reference sequence). As such, greater confidence can be assigned to larger data points and less confidence to smaller data points. The average sequencing coverage for the C28311T, A28330G, and C28312T mutations were, respectively: 9342, 8586, and 9147 reads. Therefore, confidence in the frequency estimates for these mutations is high. The data indicate that the C28311T mutation is present in effectively all samples, which is consistent with the dominant circulating lineage being Omicron. The A28330G mutation was introduced with BA.5 sub-lineages and was shown in the Ottawa data to be associated with lower sensitivity of the N1 assay. The provincial sequencing data indicate that this mutation exceeded an average frequency of 10% on June 5, 2022 which is the same date that the 14-day moving average of the Z-score reached its peak, after which it began consistently declining for several months (Fig. 5). The frequency of the A28330G mutation peaked at around 60% during the months of September and October 2022, after which it began a decline with the emergence of BQ-like sub-lineages where this mutation is absent. BQ-like sub-lineages have the C28312T mutation in addition to the C28311T mutation which is present in all Omicron sub-lineages. The frequency of the C28312T first exceeded an average of 10% on October 10, 2022 and began declining in January 2023, consistent with the initial growth of XBB-like sub-lineages in which the C28312T mutation is absent. The increase in Z-scores, occurring from early January, 2023, is consistent with the continuted decline of the A28330G mutation and the peaking of the C28312T mutation in early 2023.

**Figure 7:**
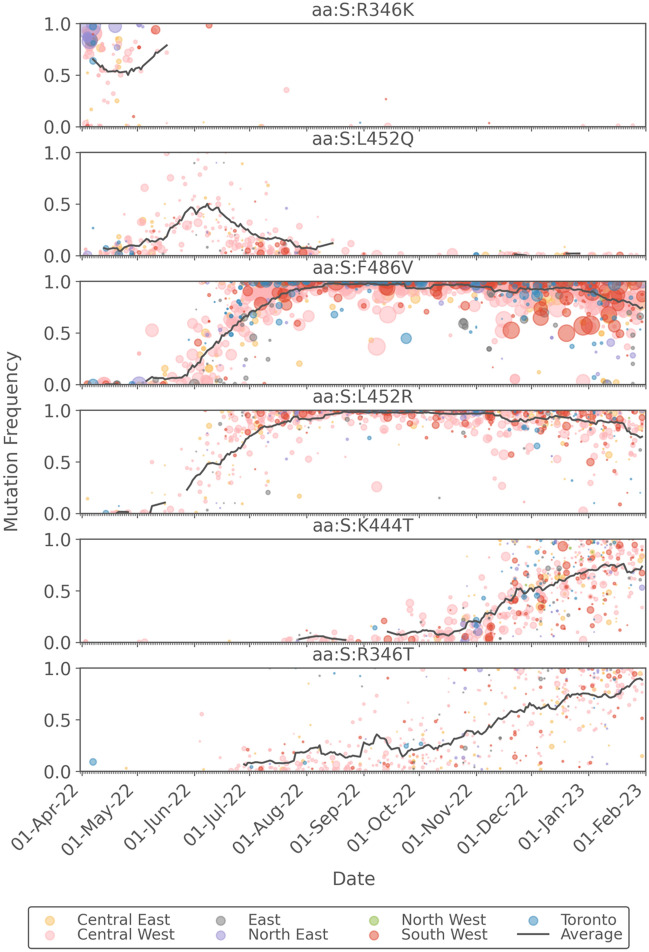
Plot of the frequencies of key mutations in the S gene. Data points are coloured by the Ontario sub-region from which the sample was taken and are sized in proportion to the sequence coverage. The solid black line represents the 14-day moving average of the daily-averaged frequencies across all sampling sites.

Further information on the sub-lineages present at the provincial level is provided through information on the S gene mutations shown in Fig. 7. The sequence coverage was uniformly lower for the spike mutations as compared to those in the N region, as a result of its presumed lower stability in wastewater. The average sequence coverages were 713 (S:R346K), 311 (S:L452Q), 963 (S:F486V), 248 (S:L452R), 250 (S:K444T), and 100 (S:R346T) reads. The S:R346K mutation is present in BA.1.1 and its sub-lineages, which the data show is present mainly before mid-May 2022. The S:L452Q mutation is present in BA.2.12.1 and peaked in early June 2022. The mutations S:F486V and S:L452R are associated with BA.4- and BA.5-like sub-lineages (including BQ-like sub-lineages), which the data show emerging in June 2022 and being present in nearly all samples from August 2022 until a decline began in January 2023, since these mutations are not present in XBB sub-lineages. The S:K444T mutation is present in BQ.1 and its sub-lineages, but is not present in BF.7 or XBB sub-lineages. The S:R346T mutation is present BQ.1 sub-lineages but is also present in BF.7 and XBB-like sub-lineages. The observation that the frequency of S:K444T peaks in January 2023 while the frequency of S:R346T continues to increase supports the notion that XBB and/or BF.7 sub-lineages began to emerge at this time. Since XBB sub-lineages have only the C28311T mutation in the N region (and not the A28330G or C28312T mutations), the apparent recovery of the N1 signal at the provincial level can be explained.

## Discussion

This study provides an example of how robust QA/QC and, specifically, a quantitative dual-loci QA/QC criteria applied to the measurement of a rapidly mutating disease target identifies loss of sensitivity of a wastewater surveillance assay affected by local mutation. Subsequently, the identification of a loss of sensitivity of the N1 assay led us to investigate the discrepancy between N1 and N2 concentrations following the emergence of Omicron sub-lineages. Basically, all COVID-19 diagnostic targets have undergone mutations and the N gene, targeted by commonly used primer/probe assays for diagnosing COVID-19, exhibits some of the highest number of mutations across the viral genome^29^. A study from Wisconsin, USA first reported diminished fluorescence intensity in SARS-CoV-2 positive-wastewater samples as proportion of Omicron infections rose when quantifying N1 gene with droplet digital PCR^9^. This finding raised concerns of possible underestimation of N1 copies during Omicron circulation when using a platform such as qPCR that solely depends upon fluorescence intensity. A study from Arkansas, USA also documented low S and N gene copies during the rise in COVID-19 waves predominated by Delta and Omicron, respectively, while quantifying SARS-CoV-2 in wastewater with three assays (S, N, and ORF1ab) using qPCR^7^. Despite the concerns and worldwide implementation of WWS for COVID-19, no quantitative QA/QC criterion pertaining to underestimation of SARS-CoV-2 RNA due to mutations had been developed. This study presents simple and necessary quantitative criteria for monitoring underestimation due to mutations that can be easily applied in laboratories worldwide implementing, at minimum, two different assays. It is recommended to implement the use of multiple assays targeting different regions of the viral genome to gain accurate results with dual loci criteria. A limitation of the approach outlined is that it may not be able to detect a change in relative signal accuracy in the unlikely event that mutations with a similar effect on signal accuracy occurred simultaneously in both loci. As a result, it is imperative that environmental laboratories track SARS-CoV-2 variants continuously, specifically monitoring for mutations in the primer/probe binding regions through genomic sequencing, to assess the impact of mutations on PCR assays and design novel assays as required.

Output from dPCR platforms (droplet- or chamber-based) is generally comparable to qPCR, although the former may offer better analytical sensitivity in WWS applications at the expense of narrower dynamic range and higher operating costs (both of these disadvantages are context-dependent). This sensitivity advantage is, at least in part, due to smaller observed matrix inhibition effects on RT and/or PCR steps^30–33^. The extent to which mutations, or groups of mutations, might differentially affect copy numbers generated by probe-based dPCR vs. qPCR has not been systematically assessed in wastewater applications. Notwithstanding this, it is expected that some mutations in the N1 or N2 gene regions that are deleterious to a qPCR signal would also affect the number of positive dPCR droplets/chambers. Moreover, changes in the quality of the sample, reagents, or operator could alter the N1/N2 ratio regardless of PCR platform. For these reasons, a straight-forward and universal (platform-independent) QC metric such as the z-score of two measurements per target is recommended as part of a routine quality assurance in a WWS program.

Previous studies on the effect of nucleotide mismatch between template and primer/probe on PCR amplification have revealed that the likelihood of pronounced effect depends upon the position and number of mismatches^34,35^. For example, multiple mismatches in the probe and in the 3′ end of primers are likely to have detrimental impact on amplification. The underestimation of SARS-CoV-2 RNA by the N1 assay in this study can be largely attributed to the mutation at position 28,330 in the probe region. Regardless, studies on the role and significance of each individual mutation present in Omicron sub-lineages on assay sensitivity are recommended in the future. During the pandemic, many customized primer/probe assays to detect SARS-CoV-2 have become commercially available to meet the growing demand for diagnostics and research applications. One limitation of using such assays is the inability to assess the impact of mutations on the sensitivity as the sequences of primers/probes are considered proprietary information and not disclosed by the manufacturer. A previous study on sensitivity of commercial assays to detect different strains of norovirus reported high cycle threshold value for one strain but failed to detect other strains, indicating that commercial assays can be designed targeting only one particular strain^36^. These findings caution against the quantification of SARS-CoV-2 based solely on commercially available assays.

The qPCR technology provides absolute quantification of targets, but to do so requires a standard curve generated from analysis of a reference DNA molecule. The reliability of an external DNA standard along with the quality of standard curve attested by the linearity and slope, can also affect the estimation of a target gene^3,37^. Despite the variation in standard curve parameters, the observed variation in SARS-CoV-2 RNA quantification by N1 and N2 assays cannot be explained by the variation of standard curve parameters. One of the limitations of the study is the inability to determine the role of fragmented nucleic acid in causing the underestimation of SARS-CoV-2 in the sludge samples tested. Viral genomes are prone to fragmentation in wastewater^38^, which can also cause discrepancies in quantification of genes of the same target organism. Fragmented genomes can also cause loss of coverage in sequencing assays, which may have affected mutation frequencies in this study. Currently, no guidelines on acceptable differences between SARS-CoV-2 RNA quantified using different assays exist. Additional studies on determining the expected difference using different SARS-CoV-2 specific assays are required.

## Conclusions

In conclusion, it has been demonstrated that an underestimation of SARS-CoV-2 N1 copy number, using the CDC N1 assay, was detected in real-time by implementing robust and quantitative dual loci QA/QC criteria for samples collected in the City of Ottawa. The proposed Z-score metric was retrospectively applied across the sampling program in the Canadian Province of Ontario where it was confirmed that this criterion could be generalized across many sites. The cause of the loss of assay sensitivity is concluded to be caused by nucleotide mismatch in the probe region; this hypothesis was supported by sequencing data which showed high proportions of the C28312T and A28330G mutations in wastewater during time periods where the accuracy was most affected. Data also showed recovery in signal accuracy as sub-lineages without the A28330G emerged toward the end of the study period, indicating that this mutation may have the most significant effect on assay sensitivity. This study highlights the importance of quantitative dual loci QA/QC criteria to continually evaluate, in near real-time, the accuracy of the signal produced in wastewater surveillance applications that rely on detection of pathogens whose genomes undergo high rates of mutation.

### Supplementary Information

Supplementary Information.

## Data Availability

The datasets generated during and/or analysed during the current study are available from the corresponding author on reasonable request.
