# Supplementary material for: Real-time evaluation of signal accuracy in wastewater surveillance of pathogens with high rates of mutation
Source: Sci Rep. 2024 Feb 14;14:3728. doi: 10.1038/s41598-024-54319-y (PMC10866965; doi:10.1038/s41598-024-54319-y)
Supplement: Supplementary file 1 — Supplementary Information. [file 41598_2024_54319_MOESM1_ESM.docx]

**Table S1: Primer and probe sequences for AS RT-qPCR assays of Omicron sub-lineages.**

| **Assay name (Targeted allele at amino acid level indicated)** | **Forward primer sequence**  **(5’-3’; lowercase indicates synthetic mismatch)** | **Reverse primer sequence (5’-3’)** | **Probe sequence and chemistry (5’-3’)** |
| --- | --- | --- | --- |
| S:L452* | GGTTGGTGGTAATTATAATTcCCT | CCGGCCTGATAGATTTCAG | FAM-TAGGAAGTCTAATCTCA-MGBNFQ |
| S:L452Q* | GGTTGGTGGTAATTATAATTcCCA |  |  |
| S:L452R* | GGTTGGTGGTAATTATAATTcCCG |  |  |
| S:K444 | CTTGGAATTCTAACAAGCTTGATTaTAA |  |  |
| S:K444T | CTTGGAATTCTAACAAGCTTGATTaTAC |  |  |
| S:L452.2 | GGTTGGTGGTAATTATAATTcCCT (same as S:L452 assay) |  | FAM-AGATTGTTTAGGAAGTCT-MGBNFQ |
| S:L452R.2 | GGTTGGTGGTAATTATAATTcCCG  (same as S:L452R assay) |  |  |
| S:K444.2 | CTTGGAATTCTAACAAGCTTGATTaTAA (same as S:K444 assay) |  |  |
| S:K444T.2 | CTTGGAATTCTAACAAGCTTGATTaTAC (same as S:K444T assay) |  |  |
| S:R346/T (duplex probe) | TaqMan™ Microbiome Mutation Assays; Assay Name: S.R346T.AGA.ACA (proprietary sequences and chemistry) | | |

* Assays modified from Wurtzer et al., 2022 (<https://doi.org/10.1016/j.scitotenv.2022.157740>) for AS RT-qPCR at this locus in the context of Omicron.

**Table S2: gBlock sequences for AS RT-qPCR**

| **Assay** | **Sequence (5’-3’)** |
| --- | --- |
| S:L452 & S:L452.2 | AAGGTTGGTGGTAATTATAATTACCTGTATAGATTGTTTAGGAAGTCTAATCTCAAACCTTTTGAGAGAGATA  TTTCAACTGAAATCTATCAGGCCGGTAGCACAGACCCCAAAATCAGCGAAATGCACCCCGCATTACGTTTGGT  GGACCCTCAGATTCAACTGGCAGTAACCAGA |
| S:L452Q &  S:L452Q.2 | AAGGTTGGTGGTAATTATAATTACCAGTATAGATTGTTTAGGAAGTCTAATCTCAAACCTTTTGAGAGAGATAT  TTCAACTGAAATCTATCAGGCCGGTAGCACAGACCCCAAAATCAGCGAAATGCACCCCGCATTACGTTTGGTG  GACCCTCAGATTCAACTGGCAGTAACCAGA |
| S:L452R &  S:L452R.2 | AAGGTTGGTGGTAATTATAATTACCGGTATAGATTGTTTAGGAAGTCTAATCTCAAACCTTTTGAGAGAGATA  TTTCAACTGAAATCTATCAGGCCGGTAGCACAGACCCCAAAATCAGCGAAATGCACCCCGCATTACGTTTG  GTGGACCCTCAGATTCAACTGGCAGTAACCAGA |
| S:K444 &  S:K444.2 | CTTGGAATTCTAACAAGCTTGATTCTAAGGTTGGTGGTAATTATAATTACCTGTATAGATTGTTTAGGAAGTCT  AATCTCAAACCTTTTGAGAGAGATATTTCAACTGAAATCTATCAGGCCGGGACCCCAAAATCAGCGAAATGC  ACCCCGCATTACGTTTGGTGGACCCTCAGATTCAACTGGCAGTAACCAGA |
| S:K444T &  S:K444T.2 | CTTGGAATTCTAACAAGCTTGATTCTACGGTTGGTGGTAATTATAATTACCTGTATAGATTGTTTAGGAAGTCT  AATCTCAAACCTTTTGAGAGAGATATTTCAACTGAAATCTATCAGGCCGGGACCCCAAAATCAGCGAAATGC  ACCCCGCATTACGTTTGGTGGACCCTCAGATTCAACTGGCAGTAACCAGA |
| S:R346 | AAATGTCTGATAATGGACCCCAAAATCAGCGAAATGCACCCCGCATTACGTTTGGTGGACCCTCAGATTCA  ACTGGCAGTAACCAGAATGGTTTTACATTCAACTCAGGACTTGTTCTTACCTTTCTTTTCCAATGTTACTTGG  TTCCATGCTATACATGTCTCTGGGACCAATGGTACTAAGAGGTTTGATAACCCTGTCCTACCATTTAATACC  ACAAAAACAACAAAAGTTGGATGGAAAGTGAGTTCAGAGTTTATTCTAGTGCGAATAATTGCACTTTTGAA  TATGTCTCTCAGCCTTGAAATCCTTCACTGTAGAAAAAGGAATCTATCAAACTTCTAACTTTAGAGTCCAAC  CAACAGAATCTATTGTTAGATTTCCTAATATTACAAACTTGTGCCCTTTTGATGAAGTTTTTAACGCCACCA  GATTTGCATCTGTTTATGCTTGGAACAGGAAGAGAATCAGCAACTGTGTTGCTGATTATTCTGTCCTATATA  ATTTCGCACCATTTTTCGCTTTTAAGTGTTATGGAGTGTCTCCTACTAAATTAAATGATAGGCTGCGTTATAGC  TTGGAATTCTAACAAGCTTGATTCTAAGGTTGGTGGTAATTATAATTACCGGTATAGATTGTTTAGGAAGTCT  AATCTCAAACCTTTTGAGAGAGATATTTCAACTGAAATCTATCAGGCCGGTAACAAACCTTGTAATGGTGTTG  CAGGTGTTAATTGTTACTTTCCTTTACAATCATATGGTTTCCGACCCACTTATG |
| S:R346T | AAATGTCTGATAATGGACCCCAAAATCAGCGAAATGCACCCCGCATTACGTTTGGTGGACCCTCAGATTCAAC  TGGCAGTAACCAGAATGGTTTTACATTCAACTCAGGACTTGTTCTTACCTTTCTTTTCCAATGTTACTTGGTTCC  ATGCTATCTCTGGGACCAATGGTACTAAGAGGTTTGATAACCCTGTCCTACCATTTAATACCACGAAAACAAC  AAAAGTCGGATGGAAAGTGAGTTAAGAGTTTATTCTAGTGCGAATAATTGCACTTTTGAATATGTCTCTCAG  CCTTGAAATCCTTCACTGTAGAAAAAGGAATCTATCAAACTTCTAACTTTAGAGTCCAACCAACAGAATCTA  TTGTTAGATTTCCTAATATTACAAACTTGTGCCCTTTTGATGAAGTTTTTAACGCCACCACATTTGCATCTGTT  TATGCTTGGAACAGGAAGAGAATCAGCAACTGTGTTGCTGATTATTCTGTCCTATATAATTTCGCACCATTTT  TCGCTTTTAAGTGTTATGGAGTGTCTCCTACTAAATTAAATGATCAGGCTGCGTTATAGCTTGGAATTCTAAC  AAGCTTGATTCTAAGGTTAGTGGTAATTATAATTACCTGTATAGATTGTTTAGGAAGTCTAAGCTCAAACCTT  TTGAGAGAGATATTTCAACTGAAATCTATCAGGCCGGTAACAAACCTTGTAATGGTGTTGCAGGTTTTAATTG  TTACTTTCCTTTACAATCATATGGTTTCCGACCCACTTATGG |

**Table S3. Standard curve performance of AS RT-qPCR assays. Values are the calculated mean of N 5-point curves.**

|  | **S:L452** | **S:L452.2** | **S:L452Q** | **S:L452R** | **S:L452R.2** | **S:K444** | **S:K444.2** | **S:K444T** | **S:K444T.2** | **S:R346** | **S:R346T** |
| --- | --- | --- | --- | --- | --- | --- | --- | --- | --- | --- | --- |
| **Slope** | -3.19 | -3.22 | -3.22 | -3.33 | -3.14 | -2.86 | -3.30 | -2.83 | -3.30 | -3.19 | -3.23 |
| **Y**-**intercept** | 35.17 | 35.46 | 35.00 | 35.75 | 35.39 | 33.97 | 38.40 | 33.91 | 38.73 | 38.65 | 39.37 |
| **R^2^** | 0.972 | 0.997 | 0.998 | 0.980 | 0.999 | 0.996 | 0.996 | 0.997 | 0.996 | 0.981 | 0.995 |
| **%Efficiency** | 113.1 | 109.7 | 104.4 | 97.67 | 103.5 | 109.3 | 99 | 108.47 | 100 | 122.6 | 102.15 |
| **N (# of curves)** | 24 | 6 | 24 | 24 | 6 | 11 | 15 | 11 | 15 | 15 | 15 |

**Table S4. List of the Z-scores calculated over the entire study period for City of Ottawa data.**

| **Date** | **Z-score** |
| --- | --- |
| 4/8/20 | 1.87543134 |
| 4/24/20 | 4.043945339 |
| 5/5/20 | -0.701326627 |
| 5/19/20 | 1.81580299 |
| 6/2/20 | 0.904156628 |
| 6/10/20 | -2.922355991 |
| 6/16/20 | 0.898289766 |
| 6/17/20 | 1.807556984 |
| 6/19/20 | 0.308129061 |
| 6/21/20 | 2.680877633 |
| 6/23/20 | 3.96967235 |
| 6/26/20 | 1.281415563 |
| 6/27/20 | 3.240934826 |
| 6/30/20 | 0.459630422 |
| 7/12/20 | 0.170058439 |
| 7/15/20 | 0.966649937 |
| 7/17/20 | -0.094107001 |
| 7/19/20 | -0.478262235 |
| 7/21/20 | 2.6089733 |
| 7/23/20 | -0.878664129 |
| 7/25/20 | 2.452311923 |
| 7/27/20 | 0.531388215 |
| 7/29/20 | 1.249314639 |
| 7/31/20 | -0.89318609 |
| 8/4/20 | -1.119044052 |
| 8/8/20 | 0.943242527 |
| 8/10/20 | 0.722101843 |
| 8/12/20 | 3.030810013 |
| 8/14/20 | 2.396663082 |
| 8/16/20 | 0.052878637 |
| 8/18/20 | 0.71131377 |
| 8/22/20 | -0.587972895 |
| 8/24/20 | 0.62414651 |
| 8/26/20 | 1.397347607 |
| 8/28/20 | 1.458704326 |
| 8/30/20 | -0.105524771 |
| 9/1/20 | 1.376596319 |
| 9/3/20 | -0.227284553 |
| 9/5/20 | 0.430199247 |
| 9/7/20 | 0.879063657 |
| 9/10/20 | 0.235660824 |
| 9/11/20 | 0.579377206 |
| 9/12/20 | -0.703560506 |
| 9/13/20 | -0.628734211 |
| 9/14/20 | 0.018773293 |
| 9/15/20 | -0.167004452 |
| 9/16/20 | -0.609412308 |
| 9/17/20 | 0.28642323 |
| 9/18/20 | -1.813218986 |
| 9/19/20 | 0.209683056 |
| 9/20/20 | 0.792515982 |
| 9/21/20 | -0.172932769 |
| 9/22/20 | -0.934943675 |
| 9/23/20 | -0.011815791 |
| 9/24/20 | -1.202441007 |
| 9/25/20 | -1.041379887 |
| 9/26/20 | -1.024592453 |
| 9/27/20 | -0.199140837 |
| 9/28/20 | -0.173293216 |
| 9/29/20 | -0.18438791 |
| 9/30/20 | 0.614401465 |
| 10/1/20 | -0.591470936 |
| 10/2/20 | 0.425645414 |
| 10/3/20 | 0.606161297 |
| 10/4/20 | -0.286636983 |
| 10/5/20 | -0.564688152 |
| 10/6/20 | -0.568516893 |
| 10/7/20 | -0.283088681 |
| 10/8/20 | -1.599264987 |
| 10/9/20 | -0.677681728 |
| 10/10/20 | -1.469736025 |
| 10/11/20 | -0.573793082 |
| 10/12/20 | 0.143611619 |
| 10/14/20 | 0.708380629 |
| 10/15/20 | -0.475745527 |
| 10/16/20 | -0.215056302 |
| 10/17/20 | -0.120895097 |
| 10/18/20 | -0.039093063 |
| 10/19/20 | -0.800303931 |
| 10/20/20 | -0.470818624 |
| 10/21/20 | 0.518596155 |
| 10/22/20 | 1.590021606 |
| 10/23/20 | 0.652952001 |
| 10/24/20 | 1.38696535 |
| 10/25/20 | -0.099303839 |
| 10/26/20 | 1.060517376 |
| 10/27/20 | -0.700758753 |
| 10/28/20 | -0.740857345 |
| 10/29/20 | -0.870603933 |
| 11/1/20 | -0.50340319 |
| 11/2/20 | 0.977671889 |
| 11/3/20 | 0.904359291 |
| 11/4/20 | 1.129129843 |
| 11/5/20 | 1.156655642 |
| 11/6/20 | 0.89877708 |
| 11/7/20 | 1.701681656 |
| 11/8/20 | -0.333002551 |
| 11/9/20 | -0.097641033 |
| 11/10/20 | 3.789334755 |
| 11/11/20 | -0.824981101 |
| 11/12/20 | 1.579604685 |
| 11/13/20 | 1.171429187 |
| 11/14/20 | 0.91103934 |
| 11/15/20 | -0.361075564 |
| 11/16/20 | -0.262659169 |
| 11/17/20 | -1.475502879 |
| 11/18/20 | 0.628515303 |
| 11/19/20 | 1.237912645 |
| 11/20/20 | 2.787471261 |
| 11/21/20 | -0.338645785 |
| 11/22/20 | -0.62017039 |
| 11/23/20 | 0.003808295 |
| 11/24/20 | -0.447653748 |
| 11/25/20 | -1.736859672 |
| 11/26/20 | -0.480908018 |
| 11/27/20 | -0.523555523 |
| 11/28/20 | -0.539223218 |
| 11/29/20 | -0.618463551 |
| 11/30/20 | -0.78334248 |
| 12/1/20 | -0.500517767 |
| 12/2/20 | 0.090752309 |
| 12/3/20 | -0.655587704 |
| 12/4/20 | -1.56993019 |
| 12/5/20 | -1.731584388 |
| 12/6/20 | -0.257948627 |
| 12/7/20 | 0.846348658 |
| 12/8/20 | -0.238507609 |
| 12/9/20 | 0.981648352 |
| 12/10/20 | -0.547384489 |
| 12/11/20 | -0.78636714 |
| 12/12/20 | -0.834915695 |
| 12/13/20 | 0.073814044 |
| 12/14/20 | -0.074484369 |
| 12/15/20 | 1.29692657 |
| 12/16/20 | -0.540405439 |
| 12/17/20 | -0.045332601 |
| 12/18/20 | -0.121101079 |
| 12/19/20 | -0.62669915 |
| 12/20/20 | -0.253164459 |
| 12/21/20 | -0.488298574 |
| 12/22/20 | -0.578413081 |
| 12/23/20 | 2.646251563 |
| 12/24/20 | -0.33685557 |
| 12/25/20 | 0.420813065 |
| 12/26/20 | 0.479756325 |
| 12/27/20 | -1.373546989 |
| 12/28/20 | 0.376434717 |
| 12/29/20 | -0.990948709 |
| 12/30/20 | 0.690220631 |
| 12/31/20 | 0.15909682 |
| 1/1/21 | 1.13600255 |
| 1/2/21 | 0.846333743 |
| 1/3/21 | -0.254214573 |
| 1/4/21 | 0.56366284 |
| 1/5/21 | 0.146427863 |
| 1/6/21 | -0.426063753 |
| 1/7/21 | 1.116861948 |
| 1/8/21 | 0.805831524 |
| 1/9/21 | 0.761254895 |
| 1/10/21 | -0.296753481 |
| 1/11/21 | 1.390403324 |
| 1/12/21 | -0.719964425 |
| 1/13/21 | 1.017925873 |
| 1/14/21 | 2.116219294 |
| 1/15/21 | -0.383285699 |
| 1/16/21 | 0.17347808 |
| 1/17/21 | -0.069367519 |
| 1/18/21 | 1.848582049 |
| 1/19/21 | -1.05704573 |
| 1/20/21 | -1.026546729 |
| 1/21/21 | 1.584973952 |
| 1/22/21 | 1.261171582 |
| 1/23/21 | 1.289412051 |
| 1/24/21 | 1.834955058 |
| 1/25/21 | -2.735531233 |
| 1/26/21 | 1.9130352 |
| 1/27/21 | 1.541659778 |
| 1/28/21 | -0.20929915 |
| 1/29/21 | -0.516832773 |
| 1/30/21 | -0.443065379 |
| 1/31/21 | -0.325972163 |
| 2/1/21 | -1.463446249 |
| 2/2/21 | 0.645431638 |
| 2/3/21 | 0.298688834 |
| 2/4/21 | 0.667328642 |
| 2/5/21 | -0.136071577 |
| 2/6/21 | 0.35535085 |
| 2/7/21 | 0.417855334 |
| 2/8/21 | -0.430852593 |
| 2/10/21 | 0.092326718 |
| 2/11/21 | 0.447121301 |
| 2/12/21 | 1.153981726 |
| 2/13/21 | -0.063395664 |
| 2/14/21 | 0.518491674 |
| 2/15/21 | 0.196999779 |
| 2/16/21 | 0.23602401 |
| 2/17/21 | 1.117640418 |
| 2/18/21 | -0.38467022 |
| 2/19/21 | 0.560225628 |
| 2/20/21 | -0.147151877 |
| 2/21/21 | 0.859139366 |
| 2/22/21 | 0.281985946 |
| 2/23/21 | 0.710329452 |
| 2/24/21 | 0.017422801 |
| 2/26/21 | 0.919953768 |
| 2/27/21 | -0.074442382 |
| 2/28/21 | 0.9943507 |
| 3/1/21 | 0.503116487 |
| 3/2/21 | 1.659515602 |
| 3/3/21 | 2.344761383 |
| 3/5/21 | -0.099281428 |
| 3/6/21 | 0.072051656 |
| 3/7/21 | 1.694486224 |
| 3/8/21 | 0.723964909 |
| 3/9/21 | 0.144137577 |
| 3/10/21 | 0.480320247 |
| 3/11/21 | -1.024589497 |
| 3/12/21 | 0.26281849 |
| 3/13/21 | 0.949530893 |
| 3/14/21 | 1.22513895 |
| 3/15/21 | -0.106271076 |
| 3/16/21 | 0.827091011 |
| 3/17/21 | 0.538140663 |
| 3/18/21 | 0.894140621 |
| 3/19/21 | 1.313652662 |
| 3/20/21 | 1.560315296 |
| 3/21/21 | 0.47977167 |
| 3/22/21 | 1.8750131 |
| 3/23/21 | 2.00893577 |
| 3/24/21 | -0.25817491 |
| 3/25/21 | -0.660384097 |
| 3/26/21 | -0.155527952 |
| 3/27/21 | 0.175224269 |
| 3/28/21 | 0.09492409 |
| 3/29/21 | -0.473852146 |
| 3/30/21 | 0.081651274 |
| 3/31/21 | 0.106553676 |
| 4/1/21 | 0.569759328 |
| 4/2/21 | 0.008800997 |
| 4/3/21 | -0.958785539 |
| 4/4/21 | 0.09444478 |
| 4/5/21 | -0.185003199 |
| 4/6/21 | 0.797414308 |
| 4/7/21 | 0.536200984 |
| 4/8/21 | -0.281931483 |
| 4/9/21 | 0.244716184 |
| 4/10/21 | 1.168876722 |
| 4/11/21 | 0.104140443 |
| 4/12/21 | 0.06891731 |
| 4/13/21 | 1.478404469 |
| 4/15/21 | -0.098795526 |
| 4/16/21 | 1.134400503 |
| 4/17/21 | 1.019277202 |
| 4/18/21 | -0.262520029 |
| 4/19/21 | 0.163002646 |
| 4/20/21 | 0.269075067 |
| 4/21/21 | 0.127498951 |
| 4/22/21 | -0.146730758 |
| 4/23/21 | -0.189058766 |
| 4/24/21 | 1.04136982 |
| 4/25/21 | 0.421276228 |
| 4/26/21 | 0.365847321 |
| 4/27/21 | 0.418489821 |
| 4/28/21 | 1.334988277 |
| 4/29/21 | 0.181075356 |
| 4/30/21 | -0.656999076 |
| 5/1/21 | -0.212431367 |
| 5/3/21 | 1.14144367 |
| 5/4/21 | 0.545343289 |
| 5/5/21 | -0.185752193 |
| 5/6/21 | 0.539769384 |
| 5/7/21 | 1.099083157 |
| 5/8/21 | 0.393154704 |
| 5/9/21 | 0.234102764 |
| 5/10/21 | -0.127745984 |
| 5/11/21 | 0.589983499 |
| 5/12/21 | 0.347529312 |
| 5/13/21 | 0.541094015 |
| 5/14/21 | 1.123176493 |
| 5/15/21 | 1.6712047 |
| 5/16/21 | -0.325200795 |
| 5/17/21 | 0.428811823 |
| 5/18/21 | -0.079641301 |
| 5/19/21 | 0.56464212 |
| 5/20/21 | 2.309075091 |
| 5/21/21 | 0.196603922 |
| 5/22/21 | -1.134341071 |
| 5/23/21 | 0.49403988 |
| 5/24/21 | 0.254659171 |
| 5/25/21 | 0.670969123 |
| 5/26/21 | 0.58884364 |
| 5/27/21 | -0.693777855 |
| 5/28/21 | -0.919435205 |
| 5/29/21 | -0.838456543 |
| 5/30/21 | 1.6239081 |
| 5/31/21 | 0.467598675 |
| 6/1/21 | 0.672218968 |
| 6/2/21 | -1.345167846 |
| 6/3/21 | 2.467101648 |
| 6/4/21 | 3.902611295 |
| 6/5/21 | 3.792040207 |
| 6/7/21 | -0.960137915 |
| 6/8/21 | -0.592269663 |
| 6/9/21 | -0.558854823 |
| 6/10/21 | -0.402875327 |
| 6/11/21 | 0.522145427 |
| 6/12/21 | -1.062453889 |
| 6/13/21 | 0.269738764 |
| 6/14/21 | 0.040937013 |
| 6/15/21 | 0.865489076 |
| 6/16/21 | -0.541320527 |
| 6/17/21 | 0.015880435 |
| 6/18/21 | -0.186053575 |
| 6/19/21 | 1.190004473 |
| 6/20/21 | 1.076211803 |
| 6/21/21 | -0.30482819 |
| 6/22/21 | -1.025827219 |
| 6/23/21 | 0.865489076 |
| 6/24/21 | 0.078645955 |
| 6/25/21 | -0.737745929 |
| 6/26/21 | -0.466243454 |
| 6/27/21 | 0.957467984 |
| 6/28/21 | 0.181271876 |
| 6/29/21 | 1.88794341 |
| 6/30/21 | -0.493170793 |
| 7/1/21 | -0.439570147 |
| 7/2/21 | -0.987228928 |
| 7/3/21 | 0.367688077 |
| 7/4/21 | 0.396729099 |
| 7/5/21 | -0.291851829 |
| 7/6/21 | 0.308537038 |
| 7/7/21 | -1.440115551 |
| 7/8/21 | -0.660278692 |
| 7/9/21 | 0.865489076 |
| 7/10/21 | 1.910612052 |
| 7/11/21 | 0.902756392 |
| 7/12/21 | -0.100289458 |
| 7/13/21 | 0.453773964 |
| 7/14/21 | -0.982969806 |
| 7/15/21 | -0.37397495 |
| 7/16/21 | 0.793836999 |
| 7/17/21 | 1.278360691 |
| 7/18/21 | 0.736444041 |
| 7/19/21 | -0.837514341 |
| 7/20/21 | 0.892174315 |
| 7/21/21 | 0.453773964 |
| 7/22/21 | -1.440115551 |
| 7/23/21 | -0.493170793 |
| 7/24/21 | -0.138066509 |
| 7/25/21 | -0.157158137 |
| 7/26/21 | -0.005564911 |
| 7/27/21 | -0.100289458 |
| 7/28/21 | 1.289313456 |
| 7/29/21 | 1.006895902 |
| 7/30/21 | 3.32420026 |
| 7/31/21 | -0.603494454 |
| 8/1/21 | -0.575513816 |
| 8/2/21 | -0.899004261 |
| 8/3/21 | 0.132775741 |
| 8/4/21 | 1.539719787 |
| 8/5/21 | 0.776963301 |
| 8/6/21 | 1.002005139 |
| 8/7/21 | 2.032015226 |
| 8/8/21 | 0.217037775 |
| 8/9/21 | -0.37397495 |
| 8/10/21 | 0.292362926 |
| 8/11/21 | 0.88885669 |
| 8/12/21 | -0.284669195 |
| 8/13/21 | -0.883514434 |
| 8/14/21 | -0.507966805 |
| 8/15/21 | 0.412422665 |
| 8/16/21 | 0.308537038 |
| 8/17/21 | -0.361038967 |
| 8/18/21 | -0.335346667 |
| 8/19/21 | -0.433986746 |
| 8/20/21 | -2.17662814 |
| 8/21/21 | 0.668988682 |
| 8/22/21 | 0.481790081 |
| 8/23/21 | -0.27464508 |
| 8/24/21 | 0.56388382 |
| 8/25/21 | 0.957467984 |
| 8/26/21 | 0.051933938 |
| 8/27/21 | 0.104050048 |
| 8/28/21 | 1.699753908 |
| 8/29/21 | 0.023344529 |
| 8/30/21 | -0.259677565 |
| 8/31/21 | -0.11439289 |
| 9/1/21 | -0.525824061 |
| 9/2/21 | 0.132775741 |
| 9/3/21 | -0.186053575 |
| 9/4/21 | -0.660278692 |
| 9/5/21 | -0.297251188 |
| 9/6/21 | 0.902756392 |
| 9/7/21 | -1.529691406 |
| 9/8/21 | -0.186053575 |
| 9/9/21 | -0.811540151 |
| 9/10/21 | 0.56388382 |
| 9/11/21 | 0.009893609 |
| 9/12/21 | 0.132775741 |
| 9/13/21 | -0.874258318 |
| 9/14/21 | 0.312810209 |
| 9/15/21 | 0.769422217 |
| 9/16/21 | 0.668988682 |
| 9/17/21 | 0.76047672 |
| 9/18/21 | -1.004324365 |
| 9/19/21 | 0.84833428 |
| 9/20/21 | 2.113367869 |
| 9/21/21 | 0.161178674 |
| 9/22/21 | 0.902756392 |
| 9/23/21 | -0.335346667 |
| 9/24/21 | 1.314632834 |
| 9/25/21 | 1.130163077 |
| 9/26/21 | 1.811657012 |
| 9/27/21 | -0.611538888 |
| 9/28/21 | 1.8741911 |
| 9/29/21 | 0.401165922 |
| 9/30/21 | 0.217037775 |
| 10/1/21 | 0.35309962 |
| 10/2/21 | 0.560050815 |
| 10/3/21 | -0.078243358 |
| 10/4/21 | 0.423074062 |
| 10/5/21 | 0.74561725 |
| 10/6/21 | -2.795632238 |
| 10/7/21 | 0.79364597 |
| 10/8/21 | 0.01299956 |
| 10/9/21 | -0.454199189 |
| 10/10/21 | 0.00937762 |
| 10/11/21 | 0.500076478 |
| 10/12/21 | 0.595605057 |
| 10/13/21 | -0.671045254 |
| 10/14/21 | 1.49903392 |
| 10/15/21 | 0.251356304 |
| 10/16/21 | -0.861213001 |
| 10/17/21 | 0.438212063 |
| 10/18/21 | 0.429704451 |
| 10/19/21 | 0.425590305 |
| 10/20/21 | -0.13913361 |
| 10/21/21 | -0.692405098 |
| 10/22/21 | -0.084993012 |
| 10/23/21 | -0.54242027 |
| 10/24/21 | -1.22881241 |
| 10/25/21 | 1.30098228 |
| 10/26/21 | -0.443730505 |
| 10/27/21 | 1.012981853 |
| 10/28/21 | 0.763881841 |
| 10/29/21 | 1.733040235 |
| 10/30/21 | -0.939858752 |
| 10/31/21 | -0.091323966 |
| 11/1/21 | -0.555594486 |
| 11/2/21 | -2.723921704 |
| 11/3/21 | -1.67898955 |
| 11/4/21 | 1.637882676 |
| 11/5/21 | -0.420888632 |
| 11/6/21 | 1.927573349 |
| 11/7/21 | 2.891922753 |
| 11/8/21 | 1.422169559 |
| 11/9/21 | -1.270096598 |
| 11/10/21 | -0.854702339 |
| 11/11/21 | 0.544822375 |
| 11/12/21 | 0.007937437 |
| 11/13/21 | 1.110019064 |
| 11/14/21 | 1.164177687 |
| 11/15/21 | 2.039728171 |
| 11/16/21 | 1.002284873 |
| 11/17/21 | 0.449002605 |
| 11/18/21 | 0.025150003 |
| 11/19/21 | 0.096353666 |
| 11/20/21 | 0.773677538 |
| 11/21/21 | 0.310866878 |
| 11/22/21 | -0.397178684 |
| 11/23/21 | 0.365239511 |
| 11/24/21 | 1.036316307 |
| 11/25/21 | -0.566908105 |
| 11/26/21 | 2.496505927 |
| 11/27/21 | -0.633098948 |
| 11/28/21 | 0.399925344 |
| 11/29/21 | 1.268986671 |
| 11/30/21 | 0.464302463 |
| 12/1/21 | 0.477107916 |
| 12/2/21 | 0.212686777 |
| 12/3/21 | -0.130616589 |
| 12/4/21 | 0.644417802 |
| 12/5/21 | 0.982655831 |
| 12/6/21 | 0.235174413 |
| 12/7/21 | -2.073672002 |
| 12/8/21 | 1.114441947 |
| 12/9/21 | -2.503662117 |
| 12/10/21 | -0.701931977 |
| 12/11/21 | 1.444316693 |
| 12/12/21 | 0.651684282 |
| 12/13/21 | -0.081955198 |
| 12/14/21 | 0.200183477 |
| 12/15/21 | -1.586060396 |
| 12/16/21 | -0.319633462 |
| 12/17/21 | 0.091868239 |
| 12/18/21 | -0.20717202 |
| 12/19/21 | 0.324275698 |
| 12/20/21 | 0.405076926 |
| 12/21/21 | -1.41669324 |
| 12/23/21 | 0.621149301 |
| 12/24/21 | 0.865124779 |
| 12/25/21 | -0.285383023 |
| 12/26/21 | -0.66112153 |
| 12/27/21 | 0.030166363 |
| 12/28/21 | 0.886344846 |
| 12/29/21 | -0.207299012 |
| 12/30/21 | -0.166599132 |
| 12/31/21 | -0.203480446 |
| 1/1/22 | -0.343959047 |
| 1/2/22 | -1.03313327 |
| 1/3/22 | -0.363158714 |
| 1/4/22 | -0.57284962 |
| 1/5/22 | -0.048858942 |
| 1/6/22 | -0.798868423 |
| 1/7/22 | -0.729883152 |
| 1/8/22 | 0.262744368 |
| 1/9/22 | -0.888039955 |
| 1/10/22 | 0.265569775 |
| 1/11/22 | 1.260926384 |
| 1/12/22 | 0.613433901 |
| 1/13/22 | -0.415936261 |
| 1/14/22 | -0.833149722 |
| 1/15/22 | -0.792821456 |
| 1/16/22 | -0.331341025 |
| 1/17/22 | -0.25509985 |
| 1/18/22 | 0.609859076 |
| 1/19/22 | -1.126135109 |
| 1/20/22 | -0.460017072 |
| 1/21/22 | 0.174467024 |
| 1/22/22 | -0.686215714 |
| 1/23/22 | 0.0193308 |
| 1/24/22 | 2.296467058 |
| 1/25/22 | -0.432152151 |
| 1/26/22 | 0.102715836 |
| 1/27/22 | 0.313430411 |
| 1/28/22 | -0.399457833 |
| 1/29/22 | -0.414551832 |
| 1/30/22 | -0.286280184 |
| 1/31/22 | -0.048235511 |
| 2/1/22 | 0.122539285 |
| 2/2/22 | -0.305433885 |
| 2/3/22 | 0.254315748 |
| 2/4/22 | 0.742698628 |
| 2/5/22 | 0.804904194 |
| 2/6/22 | 0.400139916 |
| 2/7/22 | -1.433965419 |
| 2/8/22 | -0.814471268 |
| 2/9/22 | 0.140475387 |
| 2/10/22 | -0.310634783 |
| 2/11/22 | -0.357349767 |
| 2/12/22 | -0.207522295 |
| 2/13/22 | -0.545676631 |
| 2/14/22 | 0.621459913 |
| 2/15/22 | 0.19125649 |
| 2/16/22 | -0.498615 |
| 2/17/22 | -0.701872766 |
| 2/18/22 | 0.362070762 |
| 2/19/22 | 0.913497685 |
| 2/20/22 | -0.015919711 |
| 2/21/22 | 0.936901393 |
| 2/22/22 | -0.42836975 |
| 2/23/22 | 0.906692939 |
| 2/24/22 | -0.737163309 |
| 2/25/22 | -0.13106135 |
| 2/26/22 | 0.338954032 |
| 2/27/22 | -0.093629019 |
| 2/28/22 | 1.205647322 |
| 3/1/22 | -0.890791428 |
| 3/2/22 | -0.381206715 |
| 3/3/22 | 0.425347266 |
| 3/4/22 | -0.925132463 |
| 3/5/22 | 0.734890265 |
| 3/6/22 | -1.230440668 |
| 3/7/22 | 1.157191654 |
| 3/8/22 | 1.410166741 |
| 3/9/22 | 1.229757666 |
| 3/10/22 | -0.052255061 |
| 3/11/22 | -0.108521174 |
| 3/12/22 | 0.260825644 |
| 3/13/22 | 0.281777301 |
| 3/14/22 | -0.192943934 |
| 3/15/22 | 0.957158485 |
| 3/16/22 | -0.010185596 |
| 3/17/22 | 0.730305817 |
| 3/18/22 | 0.856692594 |
| 3/19/22 | 0.406601632 |
| 3/20/22 | 0.812588112 |
| 3/21/22 | 0.775598808 |
| 3/22/22 | -0.678644466 |
| 3/23/22 | -0.897282129 |
| 3/24/22 | -0.538497862 |
| 3/25/22 | -0.408257945 |
| 3/26/22 | -0.502135325 |
| 3/27/22 | -1.047461276 |
| 3/28/22 | -0.787076485 |
| 3/29/22 | -0.78748797 |
| 3/30/22 | 0.156781139 |
| 3/31/22 | -0.809059167 |
| 4/1/22 | -0.349771682 |
| 4/2/22 | -0.06103457 |
| 4/3/22 | -0.99383993 |
| 4/4/22 | -0.726185142 |
| 4/5/22 | 0.15727643 |
| 4/6/22 | 0.434742333 |
| 4/7/22 | -1.041499194 |
| 4/8/22 | -0.760239315 |
| 4/9/22 | -0.864129814 |
| 4/10/22 | -1.222061973 |
| 4/11/22 | -0.109924803 |
| 4/12/22 | 0.460691814 |
| 4/13/22 | -0.078013631 |
| 4/14/22 | 0.006659124 |
| 4/15/22 | 0.243394587 |
| 4/16/22 | -0.106494487 |
| 4/17/22 | -0.123701145 |
| 4/18/22 | -0.096702977 |
| 4/19/22 | 0.052029778 |
| 4/20/22 | -0.323411902 |
| 4/21/22 | 1.388261131 |
| 4/22/22 | -0.530101308 |
| 4/23/22 | -0.886057406 |
| 4/24/22 | -0.308346154 |
| 4/25/22 | -0.190119406 |
| 4/26/22 | 0.863292109 |
| 4/27/22 | -0.682447406 |
| 4/28/22 | -0.633140205 |
| 4/29/22 | -0.255372142 |
| 4/30/22 | 1.164675194 |
| 5/1/22 | 0.289032091 |
| 5/2/22 | -0.058751371 |
| 5/3/22 | 0.879174062 |
| 5/4/22 | 0.050180873 |
| 5/5/22 | -0.186088265 |
| 5/6/22 | 1.401033877 |
| 5/7/22 | 1.185837888 |
| 5/8/22 | 0.837167135 |
| 5/9/22 | 0.694391077 |
| 5/10/22 | 0.119830815 |
| 5/11/22 | 1.061761335 |
| 5/12/22 | 0.333217312 |
| 5/13/22 | 0.724247429 |
| 5/14/22 | 0.261688998 |
| 5/15/22 | -0.274444128 |
| 5/16/22 | -1.082147461 |
| 5/17/22 | 0.675523881 |
| 5/18/22 | -0.371487927 |
| 5/19/22 | 0.080200973 |
| 5/21/22 | 0.75111179 |
| 5/22/22 | 0.157953363 |
| 5/23/22 | -0.571080902 |
| 5/24/22 | 0.72865342 |
| 5/25/22 | 2.608027356 |
| 5/26/22 | -0.352867581 |
| 5/27/22 | -0.067331262 |
| 5/28/22 | -0.3983617 |
| 5/29/22 | 0.542698389 |
| 5/30/22 | -0.894435884 |
| 5/31/22 | 0.133446268 |
| 6/1/22 | 1.667073203 |
| 6/2/22 | 1.845275594 |
| 6/3/22 | 2.304156397 |
| 6/4/22 | 2.511153162 |
| 6/5/22 | 2.499240042 |
| 6/6/22 | -0.447919566 |
| 6/7/22 | -0.19462646 |
| 6/8/22 | 0.469961974 |
| 6/9/22 | -0.352895787 |
| 6/10/22 | 0.482591676 |
| 6/11/22 | -0.309201271 |
| 6/12/22 | 0.063580729 |
| 6/13/22 | 0.218041522 |
| 6/14/22 | 0.72039074 |
| 6/15/22 | -0.897593944 |
| 6/16/22 | -0.483702371 |
| 6/17/22 | 0.016071401 |
| 6/18/22 | -0.653719856 |
| 6/19/22 | -0.763906548 |
| 6/20/22 | -1.525144024 |
| 6/21/22 | 0.068182306 |
| 6/22/22 | -0.171204581 |
| 6/23/22 | -0.498005661 |
| 6/24/22 | -0.937742973 |
| 6/25/22 | -1.165587413 |
| 6/26/22 | -0.915757224 |
| 6/27/22 | -0.345166684 |
| 6/28/22 | 0.086775233 |
| 6/29/22 | -0.818162919 |
| 6/30/22 | -0.325370494 |
| 7/1/22 | 0.475904539 |
| 7/2/22 | 0.528719236 |
| 7/3/22 | 1.378063531 |
| 7/4/22 | 0.162542904 |
| 7/5/22 | 0.891117863 |
| 7/6/22 | -0.273995446 |
| 7/7/22 | -1.087950062 |
| 7/8/22 | -1.704060462 |
| 7/9/22 | -1.293319781 |
| 7/10/22 | -0.956106397 |
| 7/11/22 | 0.174140127 |
| 7/12/22 | -0.923196852 |
| 7/13/22 | -0.372683576 |
| 7/14/22 | -0.86080337 |
| 7/15/22 | -1.716131519 |
| 7/16/22 | -1.697037189 |
| 7/17/22 | -1.956401828 |
| 7/18/22 | -1.259070767 |
| 7/19/22 | -0.025427806 |
| 7/20/22 | 0.9269122 |
| 7/21/22 | -0.935509695 |
| 7/22/22 | -0.092931059 |
| 7/23/22 | -0.768013896 |
| 7/24/22 | -1.121183594 |
| 7/25/22 | -0.365385989 |
| 7/26/22 | -0.020150128 |
| 7/27/22 | -1.023344772 |
| 7/28/22 | -0.855279322 |
| 7/29/22 | -0.116357295 |
| 7/30/22 | -0.858175472 |
| 7/31/22 | -0.769026687 |
| 8/1/22 | -0.342355991 |
| 8/2/22 | -1.006057591 |
| 8/3/22 | -0.965841274 |
| 8/4/22 | 0.18141251 |
| 8/5/22 | -0.586212614 |
| 8/6/22 | -1.161447515 |
| 8/7/22 | -1.272991559 |
| 8/8/22 | -1.038444844 |
| 8/9/22 | -1.146667555 |
| 8/10/22 | -0.408819465 |
| 8/11/22 | 0.558434368 |
| 8/12/22 | 0.159844117 |
| 8/13/22 | 0.566387559 |
| 8/14/22 | 0.191376393 |
| 8/15/22 | -1.170028454 |
| 8/16/22 | -0.904914941 |
| 8/17/22 | -1.215335067 |
| 8/18/22 | -1.202585584 |
| 8/19/22 | -1.279319095 |
| 8/20/22 | -1.405488709 |
| 8/21/22 | -1.510067492 |
| 8/22/22 | -1.480893054 |
| 8/23/22 | -1.186462388 |
| 8/24/22 | -0.887005819 |
| 8/25/22 | -1.595330309 |
| 8/26/22 | -0.838202261 |
| 8/27/22 | -0.671579123 |
| 8/28/22 | -1.779783226 |
| 8/29/22 | -2.552404306 |
| 8/30/22 | -2.181004619 |
| 8/31/22 | 3.188728794 |
| 9/1/22 | -1.398436895 |
| 9/2/22 | -0.958333286 |
| 9/3/22 | -1.053147416 |
| 9/4/22 | -0.823808884 |
| 9/5/22 | -0.70659276 |
| 9/6/22 | -1.281504922 |
| 9/7/22 | -1.178144659 |
| 9/8/22 | -1.017639226 |
| 9/9/22 | -1.218525212 |
| 9/10/22 | -1.804249571 |
| 9/11/22 | -1.587892653 |
| 9/12/22 | -1.708426003 |
| 9/13/22 | -1.194506586 |
| 9/14/22 | -0.802850484 |
| 9/15/22 | -0.830043152 |
| 9/16/22 | -1.425715472 |
| 9/17/22 | -0.998269491 |
| 9/18/22 | -1.606856003 |
| 9/19/22 | -1.666161535 |
| 9/20/22 | -1.382398441 |
| 9/21/22 | -1.322440096 |
| 9/22/22 | -1.656664398 |
| 9/23/22 | -0.051678731 |
| 9/24/22 | -0.625285997 |
| 9/25/22 | -1.22085778 |
| 9/26/22 | -0.454565063 |
| 9/27/22 | -1.287456782 |
| 9/28/22 | -1.369598993 |
| 9/29/22 | -0.740330077 |
| 9/30/22 | -0.858333068 |
| 10/1/22 | -1.902865793 |
| 10/2/22 | -1.103179894 |
| 10/3/22 | -0.439070149 |
| 10/4/22 | -1.482802897 |
| 10/5/22 | -0.624942747 |
| 10/6/22 | -1.013584781 |
| 10/7/22 | -0.978202046 |
| 10/8/22 | -1.123153022 |
| 10/9/22 | -1.021831628 |
| 10/10/22 | -1.169429388 |
| 10/11/22 | -0.735582731 |
| 10/12/22 | -1.427153967 |
| 10/13/22 | -0.549553648 |
| 10/14/22 | 0.113224659 |
| 10/15/22 | 0.296084297 |
| 10/16/22 | -0.261859841 |
| 10/17/22 | 1.398970455 |
| 10/18/22 | 0.135105414 |
| 10/19/22 | 0.749801477 |
| 10/20/22 | 0.85870524 |
| 10/21/22 | 1.133458321 |
| 10/22/22 | 0.667708572 |
| 10/23/22 | 0.25185855 |
| 10/24/22 | -0.807699396 |
| 10/25/22 | 0.311907937 |
| 10/26/22 | -0.667704374 |
| 10/27/22 | -0.812089259 |
| 10/28/22 | -1.117700206 |
| 10/29/22 | -0.873303766 |
| 10/30/22 | -1.329255397 |
| 10/31/22 | -1.016934255 |
| 11/1/22 | -0.697609504 |
| 11/2/22 | -0.900242095 |
| 11/3/22 | -0.457116942 |
| 11/4/22 | -0.986588278 |
| 11/5/22 | -1.336264526 |
| 11/6/22 | -1.03156084 |
| 11/7/22 | 0.139353847 |
| 11/8/22 | 0.34110177 |
| 11/9/22 | -1.113971835 |
| 11/10/22 | -0.973106028 |
| 11/11/22 | -0.937229803 |
| 11/12/22 | -0.885749322 |
| 11/13/22 | -1.304911123 |
| 11/14/22 | 0.296694476 |
| 11/15/22 | -0.224616893 |
| 11/16/22 | -0.505090121 |
| 11/17/22 | -1.12040301 |
| 11/18/22 | 0.480625406 |
| 11/19/22 | 0.102016584 |
| 11/20/22 | 0.045459801 |
| 11/21/22 | -0.533270609 |
| 11/22/22 | 1.443118012 |
| 11/23/22 | 0.292844683 |
| 11/24/22 | -0.943134315 |
| 11/25/22 | -0.135469194 |
| 11/26/22 | -1.139081758 |
| 11/27/22 | -0.960473586 |
| 11/28/22 | -1.633642956 |
| 11/29/22 | -0.693618247 |
| 11/30/22 | -0.112474062 |
| 12/1/22 | -0.801973461 |
| 12/2/22 | -0.947648758 |
| 12/3/22 | -0.559035055 |
| 12/4/22 | -0.850265945 |
| 12/5/22 | -0.621298499 |
| 12/6/22 | 0.811998261 |
| 12/7/22 | -1.662549277 |
| 12/8/22 | -0.295277836 |
| 12/9/22 | -1.02330981 |
| 12/10/22 | -1.350405685 |
| 12/11/22 | -1.132874381 |
| 12/12/22 | 0.374292029 |
| 12/13/22 | -0.820981536 |
| 12/14/22 | -0.51030726 |
| 12/15/22 | -0.518792542 |
| 12/16/22 | -1.497941511 |
| 12/18/22 | -1.169939183 |
| 12/19/22 | 0.202787065 |
| 12/20/22 | 0.008465588 |
| 12/21/22 | -1.290159874 |
| 12/22/22 | -0.161457738 |
| 12/23/22 | -0.6270645 |
| 12/24/22 | -0.879626365 |
| 12/25/22 | -0.917315342 |
| 12/26/22 | -1.018450803 |
| 12/27/22 | -0.697967013 |
| 12/28/22 | -1.42380749 |
| 12/29/22 | -1.143794096 |
| 12/30/22 | -1.41109615 |
| 12/31/22 | -1.761784076 |
| 1/1/23 | -2.119008118 |
| 1/2/23 | -1.91779872 |
| 1/3/23 | -0.191649393 |
| 1/4/23 | 1.442705716 |
| 1/5/23 | -1.391008913 |
| 1/6/23 | -1.361396218 |
| 1/7/23 | -1.082381056 |
| 1/8/23 | -1.194087803 |
| 1/9/23 | -0.201316994 |
| 1/10/23 | -0.602018764 |
| 1/11/23 | -1.113684269 |
| 1/12/23 | -1.139580159 |
| 1/13/23 | 0.359287596 |
| 1/14/23 | -0.04293191 |
| 1/15/23 | -0.094924766 |
| 1/16/23 | -0.203802202 |
| 1/17/23 | 0.212141943 |
| 1/18/23 | 0.300604501 |
| 1/19/23 | 0.852442148 |
| 1/20/23 | 0.090651146 |
| 1/21/23 | -0.034155053 |
| 1/22/23 | 0.175800329 |
| 1/23/23 | -0.297535692 |
| 1/24/23 | 1.990300589 |
| 1/25/23 | 1.472051626 |
| 1/26/23 | 0.413950668 |
| 1/27/23 | 0.468587816 |
| 1/28/23 | 0.243831349 |
| 1/29/23 | 0.088305834 |
| 1/30/23 | -0.544725218 |
| 1/31/23 | 0.991725739 |

**Table S5. List of the public health units belonging to each sub-region.**

| **Region** | **Public Health Unit** |
| --- | --- |
| North West | - Northwestern Health Unit - Thunder Bay District Health Unit |
| North East | - Algoma Public Health - North Bay Parry Sound District Health Unit - Porcupine Health Unit - Public Health Sudbury & Districts - Timiskaming Health Unit |
| East | - Eastern Ontario Health Unit - Hastings Prince Edward Public Health - Kingston, Frontenac and Lennox & Addington Public Health - Leeds, Grenville & Lanark District Public Health - Ottawa Public Health - Renfrew County and District Health Unit |
| Central East | - Haliburton, Kawartha, and Pine Ridge District Health Unit - Peterborough Public Health - Simcoe Muskoka District Health Unit |
| Toronto (metropolitan area) | - Durham Region Health Department - Halton Region Public Health - Peel Public Health - Toronto Public Health - York Region Public Health |
| South West | - Chatham-Kent Public Health - Grey Bruce Health Unit - Huron Perth Public Health - Lambton Public Health - Middlesex-London Health Unit - Southwestern Public Health - Windsor-Essex County Health Unit |
| Central West | - Brant County Health Unit - Haldimand-Norfolk Health Unit - City of Hamilton Public Health Services - Niagara Region Public Health - Region of Waterloo Public Health and Emergency Services - Wellington-Dufferin-Guelph Public Health |


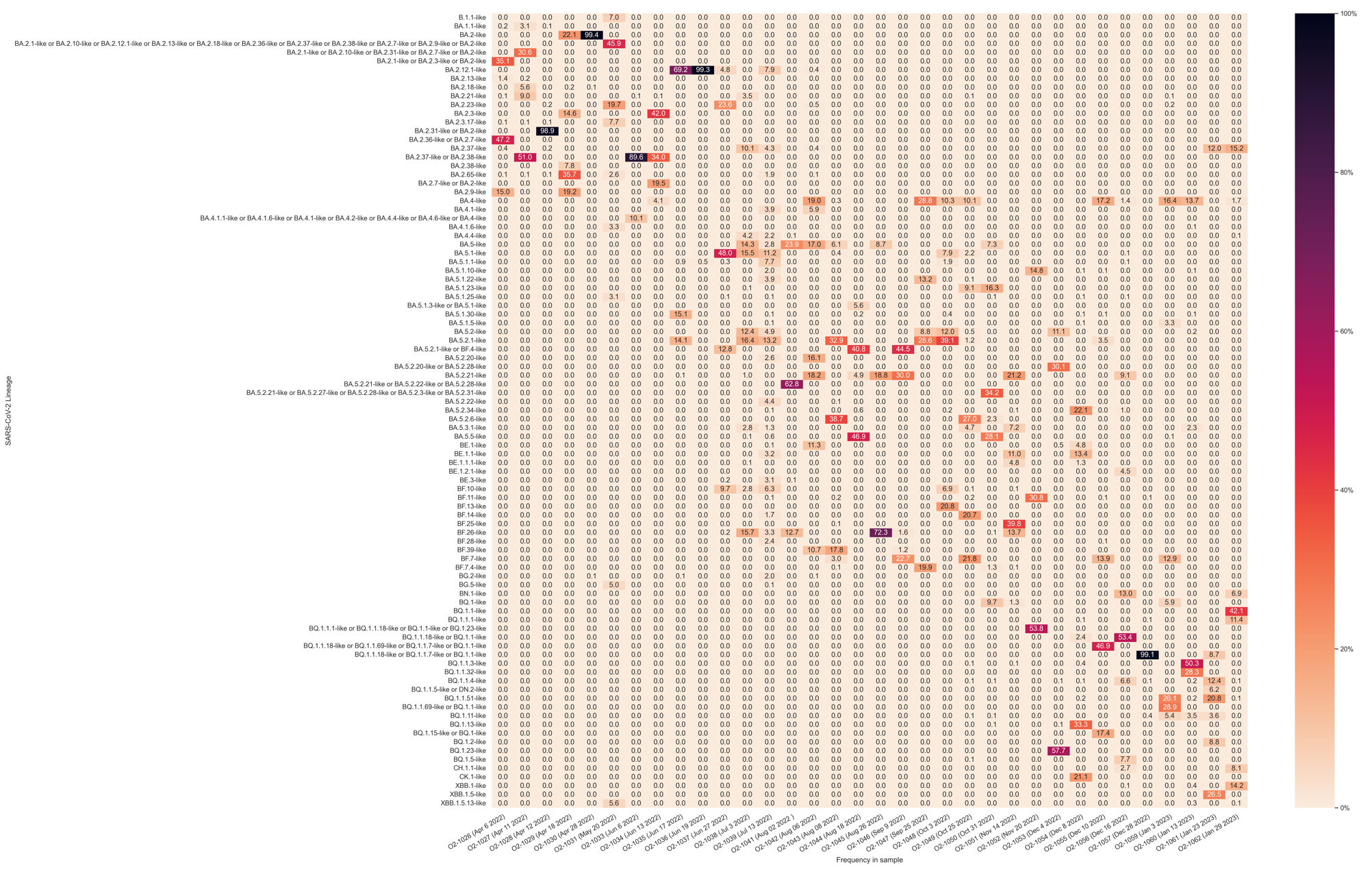


**Figure S1. Lineage frequencies for University of Ottawa from April 2022 to January 2023 as predicted by ALCOV.** The x-axis lists the wastewater samples; O2 is the site ID given to University of Ottawa Influent Post Grit, this is followed by a 4 digit internal identifier and the date each was sampled in brackets. The y-axis lists the lineages predicted by ALCOV. Lineages were included in the heatmap if predicted at 1.0% or higher in any sample on the plot. Row labels including ‘or’ signify that ALCOV was unable to distinguish between the lineages listed on that line, so any of the listed lineages may be responsible for the frequencies listed on that line. The frequencies predicted are represented out of 100% and coloured according to the legend on the right of the image where light beige represents frequencies close to 0% and black represents frequencies close to 100%. Each column representing a sample accounts for ~100% of the sample, where any missing % were assigned to lineage(s) with an abundance lower than 1%.
